# Supplementary material for: Synaptic injury in the inner plexiform layer of the retina is associated with progression in multiple sclerosis
Source: Cell Rep Med. 2024 Apr 3;5(4):101490. doi: 10.1016/j.xcrm.2024.101490 (PMC11031420; doi:10.1016/j.xcrm.2024.101490)
Supplement: Document S1. Data S1, S2, and S4 and Methods S3 [file mmc1.pdf]

Cell Reports Medicine, Volume *5*

Supplemental information

Synaptic injury in the inner plexiform layer of the retina is associated with progression in multiple sclerosis

Christian Cordano, Sebastian Werneburg, Ahmed Abdelhak, Daniel J. Bennett, Alexandra Beaudry-Richard, Greg J. Duncan, Frederike C. Oertel, W. John Boscardin, Hao H. Yiu, Nora Jabassini, Lauren Merritt, Sonia Nocera, Jung H. Sin, Isaac P. Samana, Shivany Y. Condor Montes, Kirtana Ananth, Antje Bischof, UCSF MS EPIC Team, Bardia Nourbakhsh, Stephen L. Hauser, Bruce A.C. Cree, Ben Emery, Dorothy P. Schafer, Jonah R. Chan, and Ari J. Green

```matlab
clc;    % Clear the command window.
close all;  % Close all figures (except those of imtool.)
clear;  % Erase all existing variables. Or clearvars if you want.
workspace;  % Make sure the workspace panel is showing.
format long g;
format compact;
fontSize = 20;
%===============================================================================
% Read in a standard MATLAB gray scale demo image.
folder = pwd;
%===========================================================1
%===========================================================1
%===========================================================1
%===========================================================1
%=====================|| PLEASE EDIT ONLY THE LINE BELOW! ======================================1
%========================
%===========================================================1
%===========================================================1
NameOfImage = ['IPL_central_Retina_1a_02'];        % WRITE THE NAME OF THE IMAGE!
%===========================================================1
%===========================================================1
%===========================================================1
%===========================================================1
%===========================================================1
%===========================================================1
%===========================================================1
%===========================================================1
%===========================================================1
%===========================================================1
%===========================================================1
%===========================================================1
%===========================================================1
baseFileName = strcat(NameOfImage,'.png');
% Get the full filename, with path prepended.
fullFileName = fullfile(folder, baseFileName);
% Check if file exists.
if ~exist(fullFileName, 'file')
 % The file doesn't exist -- didn't find it there in that folder.
 % Check the entire search path (other folders) for the file by stripping off the folder.
 fullFileNameOnSearchPath = baseFileName; % No path this time.
 if ~exist(fullFileNameOnSearchPath, 'file')
   % Still didn't find it.  Alert user.
   errorMessage = sprintf('Error: %s does not exist in the search path folders.', fullFileName);
   uiwait(warndlg(errorMessage));
   return;
 end
end
grayImage = imread(fullFileName);
% Get the dimensions of the image.
% numberOfColorChannels should be = 1 for a gray scale image, and 3 for an RGB color image.
[rows, columns, numberOfColorChannels] = size(grayImage);
if numberOfColorChannels > 1
 % It's not really gray scale like we expected - it's color.
 % Use weighted sum of ALL channels to create a gray scale image.
 grayImage = rgb2gray(grayImage);
 % ALTERNATE METHOD: Convert it to gray scale by taking only the green channel,
 % which in a typical snapshot will be the least noisy channel.
 % grayImage = grayImage(:, :, 2); % Take green channel.
end
% Display the image.
subplot(2, 2, 1);
imshow(grayImage);
title('Original Grayscale Image', 'FontSize', fontSize, 'Interpreter', 'None');
% Set up figure properties:
% Enlarge figure to full screen.
set(gcf, 'Units', 'Normalized', 'OuterPosition', [0, 0, 1, 1]);
% Get rid of tool bar and pulldown menus that are along top of figure.
% set(gcf, 'Toolbar', 'none', 'Menu', 'none');
% Give a name to the title bar.
set(gcf, 'Name', 'IPL Segmentation', 'NumberTitle', 'Off')
% Get a binary image of the black lines
binaryImage = grayImage > 128;
[labeledImage, numBlobs] = bwlabel(binaryImage);
% Display the image.
```

```
subplot(2, 2, 2);
imshow(binaryImage, []);
axis on;
title('Binary Image', 'FontSize', fontSize, 'Interpreter', 'None');
% Find width at every row
widths = zeros(rows, 1);
for row = 1 : rows
 thisRow = binaryImage(row, :);
 leftPixel = find(thisRow, 1, 'first');
 rightPixel = find(thisRow, 1, 'last');
 if isempty(leftPixel) || isempty(rightPixel)
   continue; % Skip lines where there is no white pixel.
 end
widths(row) = rightPixel - leftPixel; % Add 1 if you want whole pixels instead of pixel center-to-pixel
center.
end
% Display the plot.
subplot(2, 2, 3:4);
plot(widths, 'b-', 'LineWidth', 2);
grid on;
xlabel('Row', 'FontSize', fontSize);
ylabel('Width in pixels', 'FontSize', fontSize);
title('Widths', 'FontSize', fontSize);

T = table(widths);
B = rmoutliers(T, 'mean');
ResultsTable = strcat(NameOfImage,'.xlsx');
writetable(B, ResultsTable);
```

Data S1: **MATLAB code for retinal segmentation**, related to IPL segmentation, STAR methods.

Cohort				
	**RR-RR**	**RR-SP**	**p**	**Standardized Mean Difference**
n *patients*	38	19		
n *eyes*	76	37		
Fingolimod treated *N eyes, (%)*	4 (5.3)	6 (16.2)	0.026	0.109
Male sex *N eyes, (%)*	40 (52.6)	20 (54.1)	1	0.029
Baseline Age (years) *mean (SD)*	47.68 (10.17)	48.28 (10.48)	0.773	0.058
Baseline Disease Duration (years) *median [IQR]*	13.25 [9.70, 20.20]	14.30 [7.80, 19.60]	0.845	0.03
Final EDSS *median [IQR]*	2.00 [1.50, 3.00]	4.50 [3.375, 6.00]	<0.001	1.927
Study Interval (years) *mean (SD)*	3.80 (1.58)	3.43 (2.16)	0.297	0.198
Baseline Image Quality (dB) *mean (SD)*	33.42 (3.35)	33.16 (4.64)	0.736	0.064
Baseline IPL Thickness (µm) *mean (SD)*	37.00 (5.15)	35.64 (4.07)	0.16	0.294
Baseline Retinal Thickness (µm) *mean (SD)*	324.42 (18.98)	321.81 (13.93)	0.459	0.157

Data S2: **Demographics and imaging information for the full cohort**. Related to Retinal Imaging Protocol, STAR methods. Demographics, baseline age, baseline disease duration, final EDSS, study interval, image quality, baseline inner plexiform layer (IPL), and baseline retinal thickness for patients with stable relapsing-remitting disease (RR-RR) and patients who transitioned to secondary progressive on study (RR-SP). Mean and Standard deviation (SD) are represented for normally distributed variables, while median and Interquartile range (IQR) and represented for non-normally distributed variables. P values are derived from students t-test for normally distributed continuous variables, Wilcoxon-Rank Sum test for non-normally distributed variables, and Fisher's Exact test for categoric variables.

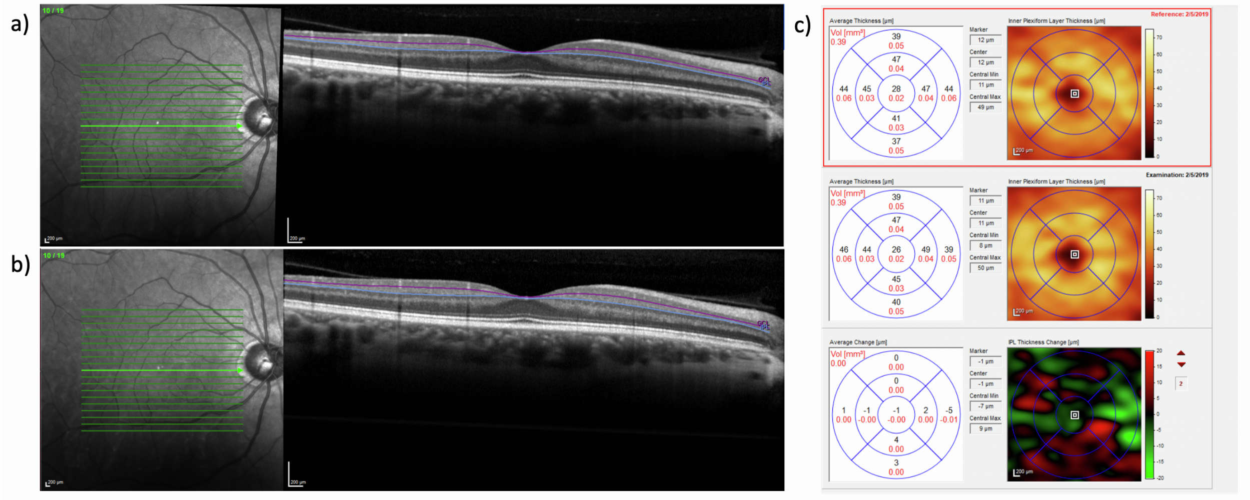

Methods S3: **Examples of segmented IPL of a single initial (a) and follow-up (b) B-scan from the test-retest analysis.** Related to Retinal Imaging Protocol, STAR methods. Panel C shows IPL thickness heatmaps across the full macular fundus, with measurement grids displaying calculated IPL volumes and the difference in measurement between the initial and follow-up volume scans.

Section	Item	Details
Inclusion criteria & Screening	Inclusion criteria	The inclusion criteria for this study are those of the parent observational study EPIC (cite). Briefly, patients qualified for EPIC if they met diagnostic criteria for CIS or multiple sclerosis. The entire EPIC cohort was screened for inclusion in this retrospective analysis. SPMS participants were selected for inclusion if they had transitioned from RRMS to SPMS following study enrollment, and had at least 2 OCT exams prior to July 1st of the year of clinical transition. A group of participants who have remained RRMS while on study were matched to participants in the SPMS group by gender, disease duration within 6 years, and age within 9 years at time of first OCT exam considered. This was done in a 2:1 control:patient matching scheme. For the primary analysis, participants were excluded if they had taken Fingolimod during the study period considered. For the secondary analysis, participants were included irrespective of Fingolimod history. At the eye level, exclusion criteria included evidence of ophthalmologic disease and failure to meet OSCARIB quality guidelines.
	Total participants screened	75 SPMS patients (all those who had transitioned on study as of Apr. 2020), 696 RRMS patients with stable disease
	SPMS Participants Excluded (reason for exclusion criteria)	56<br>(Less than 2 OCT exam dates prior to year of transition)
	Participants Excluded (reason for exclusion criteria)	658<br>(Only the 2 closest matches to the gender, age and disease duration of a SPMS patient were selected)
	Primary Analysis Participants Included (Eyes included)	16 RR-SP (31 eyes), 32 RR-RR (64 eyes)
	Secondary Analysis Participants Included (Eyes included)	19 RR-SP (37 eyes), 38 RR-RR (76 eyes)
Image Acquisition	Protocol	14 trained technicians acquired OCT images under scotopic conditions without pharmacologic dilation between Oct. 29, 2008 and Sept. 26, 2018. At each exam, 2 or more rings scans, 1 radial scan, 2 macular volume scans, and 1 posterior pole scans were acquired with the target parameters shown below. If imaging was not possible with these settings, images were acquired with reduced ART number and/or with high speed resolution settings.
	Device	Heidelberg Spectralis OCT (Model Code S2400), Heideleberg Engineering, Heideleberg, Germany
	Wavelength	870 nm
	Camera Objective	Standard
	**Acquisition Software Versions**	**Date range used (N exams)**
	3.2.1.0	10/29/2008-10/29/2008 (1)
	3.2.1.5	11/07/2008-12/01/2008 (2)
	4.0.0.0	12/08/2008-12/02/2009 (8)
	5.0.2.0	04/16/2009-11/10/2009 (7)
	5.1.2.0	12/11/2009-02/23/2010 (2)
	5.2.4.0	11/10/2010-11/10/2010 (1)
	5.3.3.0	03/16/2011-12/01/2011 (4)
	5.4.7.0	03/19/2012-08/05/2015 (48)
	5.6.3.0	05/07/2014-05/07/2014 (1)
	5.6.4.0	04/08/2013-06/25/2013 (2)
	5.7.5.0	10/23/2014-10/30/2014 (2)
	6.0.10.0	01/22/2015-08/10/2015 (6)
	6.0.13.0	08/24/2015-09/26/2018 (42)

Target Parameters

Scan Type	Anatomical Structure	Fixation Target Location	Eye Tracking	images acquired	Target Resolution	Target ART Number	Number of B-Scans, Orientation	Scan Angle (degrees)	Pattern Size (degrees)	A-scans per B-scan (HR/HS)	Depth
Ring	pRNFL	Nasal	On	>2	High Resolution	100	1 Circular	12	12 (diameter)	1536/768	1.9mm
Radial	ONH	Nasal	On	1	High Resolution	25	25 Radial	15	15 (7.5 between B-scans)	768/384	1.9mm
Macular Volume	Parafoveal macula	Middle	On	2	High Resolution	70, 16	19 Horizontal	20	20x15	1024/512	1.9mm
Posterior Pole Volume	papillomacular bundle	Middle	On	1	High Speed	10	61 Horizontal	30	30x25	768 (HS Only)	1.9mm

Section	Item	Details
Post-acquisition Analysis	Image selection	The single best (highest signal strength, ART number, and resolution setting) macular volume scan meeting OSCAR-IB quality criteria per eye and exam date was elected by a single, masked grader
	**Images reviewed** N	RR-SP: 351, RR-RR: 706
	**Primary Analysis Images selected** N (% of total reviewed)	RR-SP: 62 (18%), RR-RR: 128 (18%)
	**Secondary Analysis Images selected** N (% of total reviewed)	RR-SP: 74 (21%), RR-RR: 152 (22%)
	**Primary Analysis High Speed Images selected** N (% of total selected)	RR-SP: 6 (10%), RR-RR: 2 (2%)
	**Secondary Analysis High Speed Images selected** N (% of total selected)	RR-SP: 13 (18%), RR-RR: 2 (1%)
	**Primary Analysis ART number of selected images** median (range)	RR-SP: 68 (14-71), RR-RR: 70 (15-71)
	**Secondary Analysis ART number of selected images** median (range)	RR-SP: 68 (14-71), RR-RR: 70 (15-71)
	**Primary Analysis Signal strength**	RR-SP: 32 (5), RR-RR: 33 (3)

	(Quality) of selected images mean (SD)	
	Secondary Analysis Signal strength (Quality) of selected images mean (SD)	RR-SP: 32 (5), RR-RR: 33 (3)
	**Segmentation**	All segmentation was performed in a semi-automated fashion by a single, masked grader
	**Segmentation software**	Heyex HRA Viewing Module 6.8.3.0, Heidelberg Engineering, Heidelberg, Germany
	**Grid used for data extraction**	1, 2.22, 3.45mm circular measurement grid centered on fovea. All regions used.
	**Target structures** (units)	Intraretinal sub layers (thickness, um)
	**Statistical Analysis**	
	**Averaging/Eye selection strategy**	Average intraretinal layer thickness was calculated by multiplying regional thickness from each segment of the measurement grid by the segment's 2-dimensional area. Annualized rate of change was calculated at the eye level subtracting average thickness at the first exam, from average thickness at the second exam, and dividing by the year fraction (assuming a 365.25 day year) between the two exams. Each eye meeting OSCARIB criteria at both exams was included in analysis (the random effect of individual was included in statistical modeling to correct for violation of independence)
	**Statistical method**	Repeated measures ANOVA, Repeated measures ANCOVA

Data S4: **APOSTEL Table**. Related to Retinal Imaging Protocol, STAR methods.
