# Supplementary material for: Synaptic injury in the inner plexiform layer of the retina is associated with progression in multiple sclerosis
Source: Cell Rep Med. 2024 Apr 3;5(4):101490. doi: 10.1016/j.xcrm.2024.101490 (PMC11031420; doi:10.1016/j.xcrm.2024.101490)
Supplement: Document S2. Article plus supplemental information [file mmc2.pdf]

Cell Reports Medicine

Report

Synaptic injury in the inner plexiform layer of the retina is associated with progression in multiple sclerosis

Graphical abstract

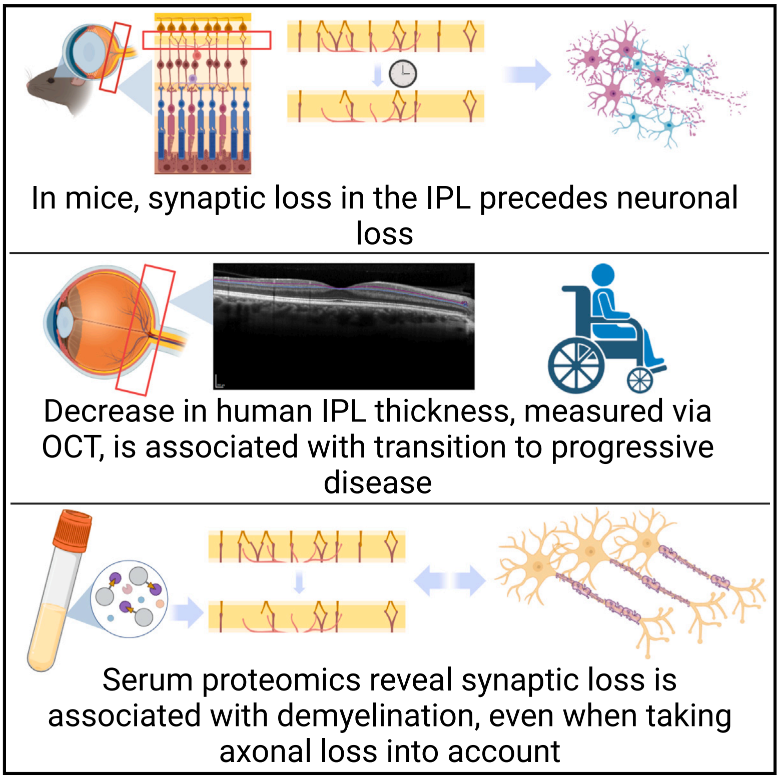

Highlights

- Synaptic injury is an early event associated with downstream demyelination in EAE
- Accelerated retinal synaptic injury precedes disease progression in people with MS
- Blood biomarkers of myelin injury and synaptic loss are independently associated

Authors

Christian Cordano, Sebastian Werneburg, Ahmed Abdelhak, ..., Dorothy P. Schafer, Jonah R. Chan, Ari J. Green

Correspondence

jonah.chan@ucsf.edu (J.R.C.),
agreen@ucsf.edu (A.J.G.)

In brief

Cordano et al. demonstrate that atrophy of the IPL (constituted by connections between ganglion cells and deeper retinal layers) could function as a biologically relevant prognostic biomarker for relentlessly progressive disease. Their work also unravels a strong correlation between demyelination and synaptic loss using blood biomarkers in an independent cohort.

Cordano et al., 2024, Cell Reports Medicine 5, 101490
April 16, 2024 © 2024 The Author(s). Published by Elsevier Inc.
https://doi.org/10.1016/j.xcrm.2024.101490

CellPress

Cell Reports Medicine

CellPress
OPEN ACCESS

Report

Synaptic injury in the inner plexiform layer of the retina is associated with progression in multiple sclerosis

Christian Cordano,[1,2,3] Sebastian Werneburg,[4,5] Ahmed Abdelhak,[1] Daniel J. Bennett,[1] Alexandra Beaudry-Richard,[1] Greg J. Duncan,[6] Frederike C. Oertel,[1] W. John Boscardin,[7] Hao H. Yiu,[8] Nora Jabassini,[1] Lauren Merritt,[4] Sonia Nocera,[1] Jung H. Sin,[1] Isaac P. Samana,[1] Shivany Y. Condor Montes,[1] Kirtana Ananth,[1] Antje Bischof,[1] UCSF MS EPIC Team[1] Bardia Nourbakhsh,[9] Stephen L. Hauser,[1] Bruce A.C. Cree,[1] Ben Emery,[6] Dorothy P. Schafer,[4] Jonah R. Chan,[1,*] and Ari J. Green[1,*]

[1]UCSF Weill Institute for Neurosciences, Department of Neurology, University of California, San Francisco, San Francisco, CA, USA
[2]Department of Neurology, Rehabilitation, Ophthalmology, Genetics, Maternal and Child Health (DINOGMI), University of Genoa, Genoa, Italy
[3]IRCCS Ospedale Policlinico San Martino, Genoa, Italy
[4]Department of Neurobiology, Brudnik Neuropsychiatric Research Institute, University of Massachusetts Chan Medical School, Worcester, MA, USA
[5]Department of Ophthalmology & Visual Sciences, Michigan Neuroscience Institute, University of Michigan – Michigan Medicine, Ann Arbor, MI, USA
[6]Jungers Center for Neurosciences Research, Department of Neurology, Oregon Health & Science University, Portland, OR, USA
[7]Department of Biostatistics, University of California, San Francisco, San Francisco, CA, USA
[8]Department of Biology, University of Maryland, College Park, MD, USA
[9]Department of Neurology, Johns Hopkins University, Baltimore, MD, USA
*Correspondence: jonah.chan@ucsf.edu (J.R.C.), agreen@ucsf.edu (A.J.G.)
https://doi.org/10.1016/j.xcrm.2024.101490

SUMMARY

While neurodegeneration underlies the pathological basis for permanent disability in multiple sclerosis (MS), predictive biomarkers for progression are lacking. Using an animal model of chronic MS, we find that synaptic injury precedes neuronal loss and identify thinning of the inner plexiform layer (IPL) as an early feature of inflammatory demyelination—prior to symptom onset. As neuronal domains are anatomically segregated in the retina and can be monitored longitudinally, we hypothesize that thinning of the IPL could represent a biomarker for progression in MS. Leveraging our dataset with over 800 participants enrolled for more than 12 years, we find that IPL atrophy directly precedes progression and propose that synaptic loss is predictive of functional decline. Using a blood proteome-wide analysis, we demonstrate a strong correlation between demyelination, glial activation, and synapse loss independent of neuroaxonal injury. In summary, monitoring synaptic injury is a biologically relevant approach that reflects a potential driver of progression.

INTRODUCTION

Despite progress in understanding and treating the immunological dysregulation underlying relapses and MRI activity, the fundamental biology of insidious disability worsening in multiple sclerosis (MS) remains poorly understood. The concept of discrete stages of relapsing and progressive disease (traditionally relapsing-remitting MS [RRMS] and secondary progressive MS [SPMS]) has recently been challenged by the observation that silent progression or progression independent of relapse occurs much earlier in the disease course than previously recognized.[1–3] Nevertheless, the concept of transition to SPMS remains clinically useful, as many relapsing-onset patients ultimately develop clinically evident insidiously progressive disease and manifest more pronounced disability during this stage of the disease. In addition, neurologists and regulatory agencies still apply a clinically based classification of relapse-onset MS into relapsing-remitting, primary, and secondary progressive stages, which guides the approval of disease-modifying treatments.

Indeed, significant differences can still be observed between RRMS and SPMS, particularly in neuropathology and responses to immunomodulatory therapy.[4–7] Hence, treatments that are effective in RRMS either have limited or no therapeutic benefit when used in progressive MS.[8] In addition, the current prevailing definition of SPMS, "progressive disability worsening that is confirmed over a period (typically 6 to 12 months) in the absence of a preceding clinical relapse," has numerous limitations;[9] it requires both meticulous relapse ascertainment as well as standardized neurological assessments performed at fixed intervals. This is particularly relevant as the evolution from RRMS to SPMS usually occurs insidiously, and it is difficult to recognize the transition until after substantial clinical disability accumulates. This lack of effective disease-modifying therapies for progression and the challenge of clinical diagnosis heightens the importance of building a strong cellular and molecular framework for progression.

This is an open access article under the CC BY license (http://creativecommons.org/licenses/by/4.0/).

Informative biomarkers are required to quantify and predict the transition into more rapid progression while being predictive of the underlying pathophysiological processes driving disease worsening. This will enhance therapeutic escalation or switching choices and enable appropriate clinical trial recruitment. Synaptic loss may be an early contributor to accumulating disability in neurodegenerative diseases.[10–12] In limited postmortem MS tissue, synaptic loss has been detected in multiple areas, including the cerebellum, neocortical areas, and the hippocampus.[13–15] In addition, demyelinated hippocampi have minimal neuronal loss but significant decreases in synaptic density. Despite this existing research, our understanding of the cellular and molecular basis of synapse loss in MS remains incomplete. This knowledge gap necessitates further investigation. Furthermore, due to their cross-sectional nature and frequent dependence on tissue obtained at the end of life, the described pathological studies cannot determine when synaptic loss occurs.

Animal models significantly improve our understanding of MS pathophysiology, and experimental autoimmune encephalomyelitis (EAE) serves as the best possible animal model for identifying potential biomarkers of progression in MS. EAE exhibits chronic inflammatory demyelination resulting in neuroaxonal loss and provides the opportunity for longitudinal neuropathological assessments. Synapse loss has been studied in both myelin oligodendrocyte glycoprotein (MOG) EAE and the toxic genetic model Plp1-CreER^{T};ROSA26-EGFP-DTA.[14,16] Synapse loss in the lateral geniculate nucleus (LGN) of the thalamus, the principal afferent target of axons from retinal ganglion cells, was found to be concomitant with reactive gliosis and activation of the alternative complement cascade in EAE. Blocking complement component C3 resulted in attenuation of the observed synapse loss and was associated with better visual performance measured by optomotor testing.[16] This suggested that synapse loss may be a significant contributor to neurological dysfunction. Therefore, it is plausible that synapse loss could represent an excellent biomarker of disease progression, but it remains unclear whether synapse loss is a precursor to degeneration of other neuronal elements in human disease (MS) or whether it precedes meaningful neurological decline.

The retina offers a unique opportunity to investigate the impact of MS on different neuronal substructures because the cell bodies, axons, and synapses are anatomically segregated.[17] Optical coherence tomography (OCT) can be used to non-invasively monitor retinal neuronal tissue loss in an area generally devoid of myelin and in a single pathway[18–22] at a much higher resolution than what is possible by brain MRI. In addition, the anatomy of the retina is relatively unique, given its organization and orientation, in that dendrites and synapses are layered in a highly predictable fashion. The inner plexiform layer (IPL) is constituted by the synapses that relay signals from bipolar cells, other cells of the inner nuclear layer, and retinal ganglion cells (RGCs). Historically, IPL tissue volume was quantitated in conjunction with the ganglion cell layer (GCL) due to challenges in reliable differentiation between the two closely opposed intra-retinal tissue layers. Technological advances in imaging technology and semi-automated intra-retinal layer border detection algorithms now allow segregation of IPL volume from the GCL volume and provide an opportunity to perform non-invasive, *in vivo* monitoring of synaptic volume in MS.

In this study, we applied a translational approach to study the association between synaptic injury and progression in MS. First, we analyzed retinal signs of synaptic injury in an animal model of inflammatory demyelination and neurodegeneration (i.e., EAE),[23] finding evidence of synaptic loss within the IPL on the first day of symptoms and prior to worsening motor outcomes. To investigate the temporal relationship between synaptic loss and relentless MS disability, we then leveraged the University of California, San Francisco (UCSF) Expressions, Proteomics, Imaging, Clinical (EPIC) dataset that follows over 800 participants enrolled with more than 12 years of average follow-up with highly detailed assessments, including OCT.[24–31] We find that people with MS (PwMS) who develop SPMS show greater IPL loss using macular volume OCT as a sign of pronounced synaptic pathology. Our findings are supported by a proteome-wide analysis from MS patients enrolled in a randomized clinical trial that explored the pathophysiological mechanisms that might drive synaptic injury in PwMS.

RESULTS

The number of intact synapses in the IPL is reduced in early EAE

EAE is an animal model of inflammatory adaptive immune-driven demyelination, most frequently induced via immunization with $MOG_{35–55}$ peptide, characterized by ascending motor impairment and visual impairment.[23,32–34] In EAE, animals exhibit ascending motor impairment and demyelinating damage to the optic nerve. The onset of motor symptoms occurs around 12 days post immunization (dpi), reaching a peak around 18 dpi. Subsequently, there is a partial recovery phase followed by a period of substantial symptom stability (Figure 1A). Previous work has established that demyelination occurs early during the disease course[35] and that demyelination within the optic nerve leads to downstream (LGN) synaptic loss.[16] Furthermore, toward the later stages of the disease, the symptoms persist chronically (Figure 1A), and neuronal loss becomes evident.[33] Here, we sought to investigate the retina in EAE as a means to identify a biomarker that can be predictive of this final outcome. We aimed to leverage the opportunity to study longitudinally a comparable biomarker in PwMS, utilizing OCT as a valuable tool for assessment. Initially, we observed a reduction in the thickness of the IPL in the retinas of EAE mice, compared with control mice, based on previously collected data. The IPL encompasses the synapses between the dendrites of the RGCs (whose axons comprise the optic nerve) and the cells contained in the inner retinal layer. Motivated by this finding, we further investigated whether demyelination could induce early upstream synaptic loss within the retina, which could serve as a marker of neuronal distress and death. EAE was induced in 20 8- to 10-week-old female C57Bl6/J mice by subcutaneous (s.c.) injection of $MOG_{35–55}$ peptide. Eyes were collected and snap-frozen from 12 mice with MOG-induced EAE (four for each time point) and 9 sham-immunized controls (three for each time point) 12, 18, and 60 dpi. The analysis was performed in the proximity of the optic disc area (up to 250 μm away from the optic disc) (Figure 1A).

Presynaptic (Bassoon and VGluT1) and postsynaptic markers (Homer 1), and the colocalization of these pre-and postsynaptic

CellPress
OPEN ACCESS

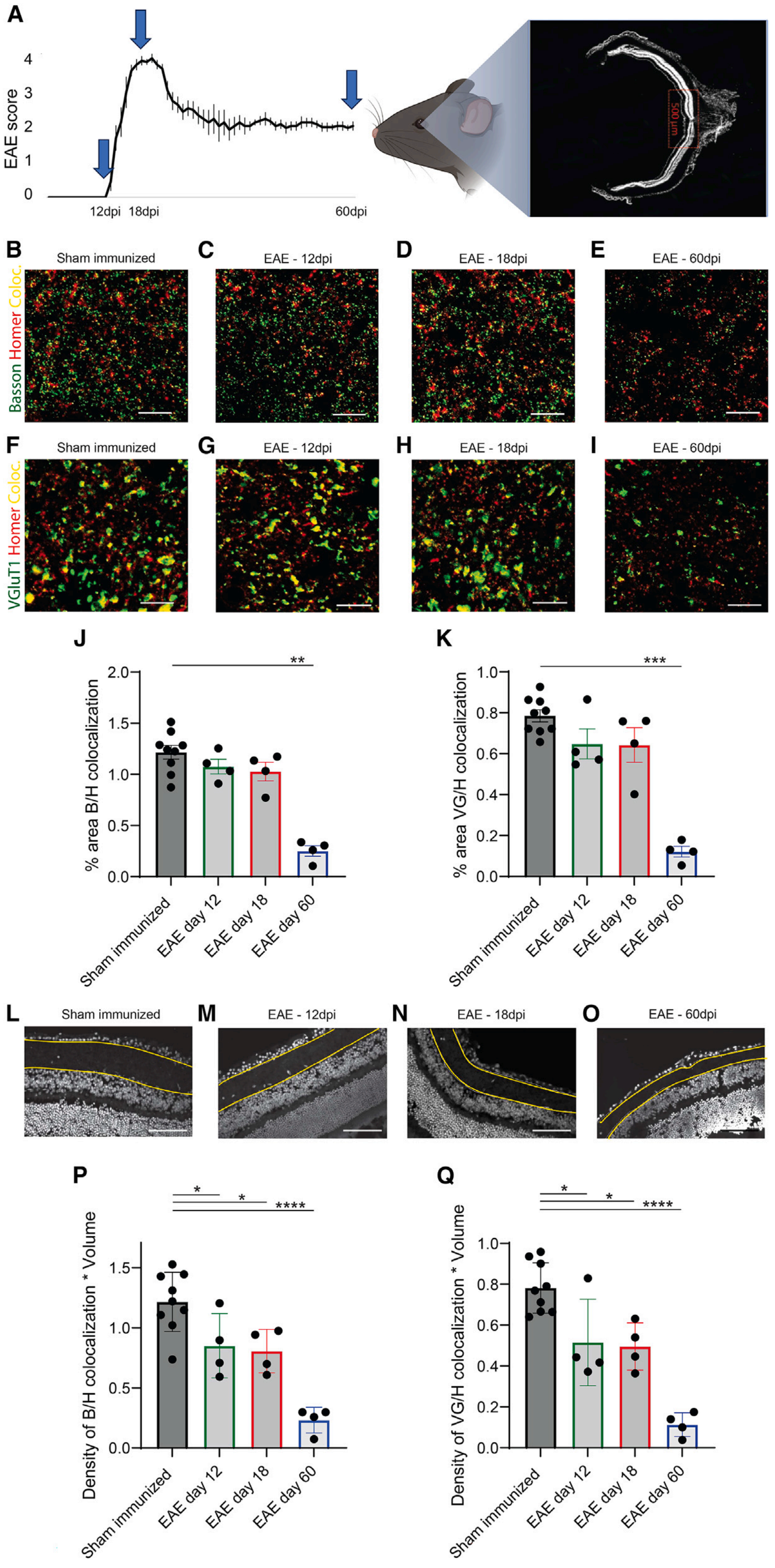

Figure 1. IPL synaptic density/volume is decreased 12 dpi

(A) Representation of an EAE score graph in a 60-day experiment (arrows indicate the timing of tissue collection). Synaptic density was calculated by analyzing the area in proximity of the optic nerve head for 12 EAE female mice and 9 sham-immunized mice at onset (12 dpi, four EAE mice and three sham-immunized mice), peak of disease (18 dpi, four EAE mice and three sham-immunized mice), and at a chronic stage (60 dpi, four EAE mice and three sham-immunized mice).

(B–I) Staining for presynaptic inputs (Bassoon and VGluT1, green) and postsynaptic compartments (Homer 1, red) and colocalization of pre-and postsynaptic terminals (yellow) in sham-immunized mice (B and F), at 12 dpi (C and G), 18 dpi (D and H), and 60 dpi (E and I). Scale bar: 10 um.

(J–K) Synaptic counts per area showed a trend toward reduction on day 12 and day 18 and on day 60 showed statistically significant synaptic loss.

(L–O) Examples of retinal IPL segmentation from DAPI images: sham-immunized mice (L), 12 dpi (M), 18 dpi (N), and 60 dpi (O). Scale bar: 100 um.

(P and Q) Quantification of the number of functional synapses per IPL volume, given by colocalization of both Bassoon/Homer (P) and VGlut1/Homer (Q) per IPL volume. *$p < 0.05$, **$p < 0.01$, ***$p < 0.001$, ****$p < 0.0001$.

Technical replicates: for each animal, 3–4 stained retinal cross-sections at the level of the optic disc containing the central retina.

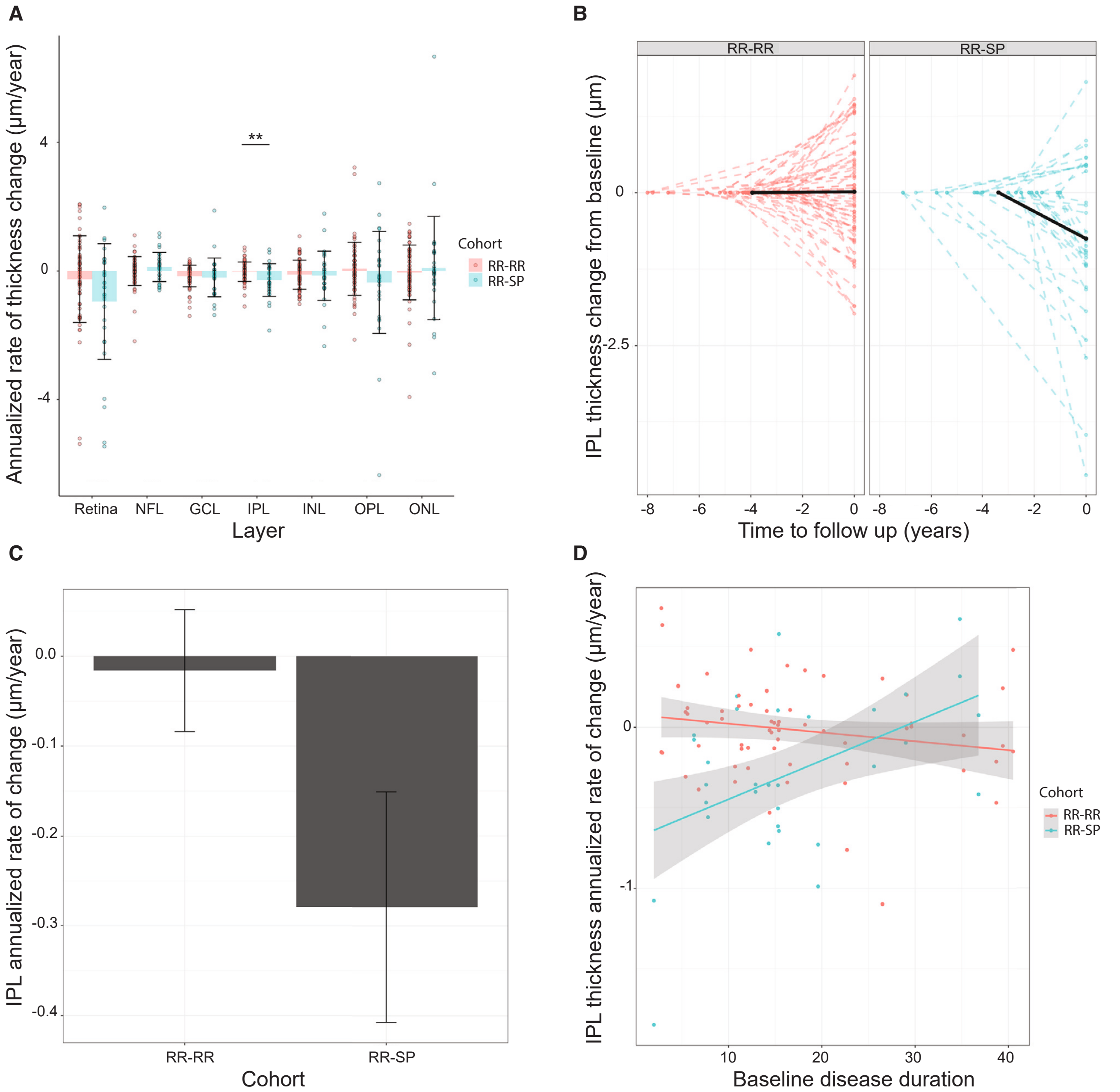

Figure 2. IPL change from baseline of the primary cohort

(A) Annualized retinal layer percentage change for the primary cohort in percent per year. Bars represent mean, whiskers represent standard deviation, and points represent data from individual eyes. Red indicates patients with a stable relapsing-remitting disease course (RR-RR), while blue indicates patients who transitioned to secondary progressive disease on the study (RR-SP). Retina, full retinal thickness; NFL, nerve fiber layer; GCL, ganglion cell layer; IPL, inner plexiform layer; INL, inner nuclear layer; OPL = outer plexiform layer; ONL, outer nuclear layer. The *p* values were derived from a linear mixed-effects model adjusted for the random effect of patients. $^{**}p < 0.01$.

(B) IPL thickness change in total microns from the baseline exam, plotted against time to follow up in years. Negative values on the y axis represent loss of IPL thickness compared with baseline measurement, while positive values represent gain. Values on the y axis represent time in years preceding follow-up exam, with the follow-up exam date set at 0. Patients with RR-RR disease are depicted in red on the left side of the plot, while patients who transitioned to secondary progressive disease (RR-SP) on the study are represented in blue on the right. Dashed lines represent single-eye data points, while solid black lines represent average cohort values.

(legend continued on next page)

CellPress
OPEN ACCESS

markers was assessed within the IPL of EAE mice and sham-immunized controls. Total synaptic counts per area field of view showed a trend toward reduction on day 12 and day 18 and on day 60 showed statistically significant synaptic loss (Figures 1B–1K). This initial analysis of density only took the average of the fields of view without taking into account the entire IPL thickness. We thus evaluated the synaptic counts correcting for changes in tissue thickness to estimate the number of functional synapses per IPL volume. For this purpose, the IPL was manually segmented on 12-μm sections stained with DAPI, and the IPL thickness was automatically measured for multiple images using MATLAB (Data S1; Figures 1L–1O). The average number of structural synapses per field of view, as indicated by the colocalization of pre-and postsynaptic terminals, was multiplied by the total IPL thickness across the entire retinal section. This correction for changes in IPL thickness showed a synaptic loss, including the period of first detectable signs of clinical symptoms at 12 dpi. This was confirmed for both the co-localization of Bassoon/Homer ($p = 0.03$; Figure 1P) and Glut1/Homer ($p = 0.01$; Figure 1Q). That is, while the average synapses per field of view showed only late decreases in synapses, taking the entire IPL into account reveals earlier synapse loss. Synaptic loss adjusted for this IPL thickness factor was also found at the peak of motor impairment (18 dpi) ($p = 0.01$ and $p = 0.002$) and became more evident at 60 dpi ($p < 0.0001$ for both co-localization of Bassoon/Homer and Glut1/Homer) (Figures 1P and 1Q). The synaptic loss detected on days 12 and 18 precedes the timing of previously described RGC loss (even accounting for variability in EAE experiments). Remarkably, using longitudinal OCT in EAE, we have also previously shown increases in the thickness of the summed total of the retinal nerve fiber layer (NFL), GCL, and IPL at 15 dpi, followed only later by thinning of the total inner retina (NFL + GCL + inner nuclear layer [INL]) at 30 dpi.[33] This would suggest that the increases in thickness around days 12–18 are largely constituted by increases in RNFL and GCL but not IPL. Our findings reveal that synaptic loss in the IPL is detectable as early as the first day of symptoms in EAE and occurs prior to the development of further disability. This led us to investigate whether the IPL could potentially serve as a predictive biomarker for disease progression in MS, using OCT.

IPL thickness is a biomarker for transition to progressive MS

Building upon our prior observations in EAE, our objective was to delve deeper into the evidence of retinal synaptic loss associated with MS. We embarked on investigating whether the potential biomarker we identified for early neuronal dysfunction in EAE could be translated and longitudinally studied in MS patients. Specifically, we aimed to ascertain whether the transition to progressive MS is preceded most prominently by axonal, somatic, or synaptic loss within the retina. We leveraged the longitudinal EPIC cohort to identify 70 PwMS who developed SPMS (RR-SP) after an initial diagnosis of RRMS at the study inclusion. Of those participants, 19 had at least two retinal imaging time points, more than 6 months apart, prior to their year of transition. An additional 38 participants with stable relapsing-remitting (RR-RR) disease were then matched to the RR-SP subjects at a 2:1 ratio based on age, sex assigned at birth, and disease duration. In summary, we included 76 and 37 eyes, respectively. Of the RR-SP patients, 54% were men and had a mean (±SD) age of 48.28 (±10.48) years and a median [interquartile range (IQR)] disease duration of 14.3 [17.8, 19.6] years at baseline. Of the RR-RR participants, 52.6% were male and 47.68 (±10.17) years old, had a disease duration of 13.25 years [9.70, 20.20] at baseline, and did not significantly differ from the RR-SP group (Data S2). Clinical features and baseline imaging characteristics, including IPL and retinal thickness (utilizing an OCT 3.45-mm parafoveal measurement circle), were comparable between the two groups (Data S2). Due to the potential impact on macular volume, retinal scans collected during treatment with fingolimod ($n = 3$ and 2 for RR-SP and RR-RR, respectively) were excluded from the primary analysis cohort. Mean (±SD) thicknesses across the whole primary analysis cohort were as follows: full retina = 324.3 μm (±17.6), NFL = 21 μm (±2.6); GCL = 40.9 μm (±7.1), IPL = 36.4 μm (5.0), INL = 37.3 μm (±3.7), outer plexiform layer (OPL) = 33.3 μm (±3.9), and outer nuclear layer (ONL) = 72.8 μm (±6.6). Baseline (first OCT) average thickness of the full retina, NFL, GCL, IPL, INL, OPL, and ONL did not differ significantly between groups.

PwMS in the RR-SP cohort lost a mean (SD) of 0.259 μm (±0.096) of IPL thickness per year, while mean thickness in the RR-RR cohort showed no changes over time ($F = 7.28$, $df = 46$, $p = 0.0097$; Figures 2A–2C). When taking into account disease-modifying treatment during follow-up, the difference between the two cohorts remained statistically significant ($p = 0.01$). Eyes with a positive history of optic neuritis had a significantly lower IPL at baseline ($p = 0.006$), but previous history of optic neuritis did not impact the results when comparing the two cohorts of PwMS ($p = 0.007$). Also, baseline age, gender, follow-up scan quality, and final EDSS did not impact the results. Moreover, the highest rate of change in the transition to progression cohort was found in subjects with a disease duration of less than 20 years ($n = 12$; Figure 2D; slope difference with $p = 0.0017$). The annualized rate of change did not differ between groups for whole retina, NFL, GCL, INL, ONL, or OPL (mixed-effects models corrected for the random effect of patients; all $p > 0.05$) (Figure 2A). The OPL, another retinal layer rich in synapses, exhibited a pattern similar to the one of the INL, albeit without attaining statistical significance due to the higher variability of the measure (Figure 2A). Our findings demonstrated that, in MS patients, the IPL thickness, a measure of *in vivo* synaptic loss, decreases prior to the diagnosis of progressive disease, whereas the other retinal layers remain relatively stable in patients who do not develop progression. These results suggest

(C) Rate of annualized (IPL) thickness change in total microns per year from the baseline exam of the primary analysis cohort (excluding patients treated with fingolimod), by disease cohort. Bars represent mean, while whiskers represent standard error (SE).
(D) Rate of annualized (IPL) thickness change in total microns per year from the baseline exam of the primary analysis cohort, by disease duration at baseline. Red represents patients with RR-RR disease, while blue represents patients who transitioned to RR-SP disease in the study. Points represent single-eye data, while lines represent general linear models. Gray borders represent the SE of the linear model fit.

that IPL loss could serve as a predictive marker for disease progression in MS. Furthermore, the observed synaptic loss highlights its potential as a key cell-level feature underlying the relentless neurological disability associated with the disease.

IPL thickness can be segmented with excellent reproducibility

Historically, the IPL was challenging to separate from the GCL using previous technological approaches. Consequently, the combined measurement of the ganglion cell and IPL (GCIPL) has been widely adopted as a proxy for the assessment of retinal neuronal loss. In light of this, we decided to establish the validity of specifically measuring IPL thickness, aiming to demonstrate its robustness as an independent measure. To demonstrate the reliability of our IPL thickness quantification, separately from the GCL, we decided to use the mean intra-eye coefficient of variation (CV). 20 participants with a median age (range) of 35.65 (23.3–57.2) years, 40% males (40 eyes), were included in a segmentation intra-rater repeatability analysis.

Mean (±SD) intra-eye CV values for measurements of full retinal thickness, GCL, and IPL were 0.002 (±0.0002), 0.0077 (±0.0005), and 0.0115 (±0.0048), respectively (Figures 3A and 3B). The CV differed significantly by layer (ANOVA; F = 40.54, df = 6, $p < 0.0001$), and the CV of the IPL was significantly higher than that of the full retina ($p < 0.001$) but did not significantly differ from that of the GCL (p = 0.64). Intra-eye absolute mean deviations for full retinal thickness, GCL, and IPL were 0.572 (0.45), 0.296 (0.39), and 0.428 (0.33) μm, respectively (Figure 3B). The maximum mean deviation for the full retina, GCL, and IPL was 1.81, 0.67, and 1.38 μm, respectively (Figure 3B). Mean deviation significantly differed between layers (ANOVA; F = 24.72, df = 6, $p < 0.0001$), but that of the IPL did not differ from that of the full retina or GCL (p = 0.07 and 0.86, respectively). There was no significant effect of eye (F = 2.96, df = 1, p = 0.086), image order (F = 1.38, df = 3, p = 0.246), or non-referenced scan (F = 0.273, df = 1, p = 0.132) on absolute mean deviation. Pearson's correlation coefficients by order of image acquisition were in the range of 0.99–0.99, 0.97–0.99, and 0.89–0.96 for full retinal thickness, GCL, and IPL, respectively. Information on additional retinal layers can be found in Figure 3B. Taken together, these results demonstrate that IPL thickness is a reliable and reproducible measure.

Defining the deriving mechanisms behind synaptic damage in MS

After establishing the temporal relationship between synaptic loss and the diagnosis of progressive disease, we decided to investigate the biological substrate of synaptic loss using a proteomics approach. We further assessed validated blood-based biomarkers that allowed us to monitor the pathophysiology at "single-protein resolution" from a unique dataset to provide independent corroboration of our findings. This evaluation also explored the correlation between glial, B, and T cell activation markers and synaptosome-associated protein of 25 kDa (SNAP-25) as a marker of synaptic damage. To that end, we conducted a hypothesis-driven analysis from a proteome-wide dataset (unpublished data) from participants with MS included in the ReBUILD trial[36] using the proximity extension assay (PEA) with the Olink explorer kit 3072 (Olink, Uppsala, Sweden) at the Olink analytical facility (Boston, MA, USA).

Specifically, we evaluated the association between an established synaptic damage marker, SNAP-25,[37,38] and blood biomarkers of the following pathophysiological processes: oligodendrocyte damage (oligodendrocyte glycoprotein [OMgp]),[39] myelin injury (MOG),[40] astrocyte activation (glial fibrillary acidic protein [GFAP]),[41] microglia activation (soluble triggering receptor expressed on myeloid cells 2 [sTREM2]),[42] astrocyte and microglia involvement (chitinase 3-like 1 [CHI3L1]),[43,44] B cell recruitment and activation (chemokine ligand 13 [CXCL-13]), and T cell activation (CD27).[45] Of note, CHI3L1 is also expressed by activated microglia,[43] which has previously been identified to contribute to neuroaxonal injury generally and synaptic engulfment specifically in MS.[16]

Our analysis included 166 longitudinal samples from 47 individuals with a mean age at the inclusion of 39.4 (±10.3) and a disease duration of 4.4 years (±3.6). None of the included participants experienced clinical or imaging disease activity or a switch of disease modifying therapy (DMT) 3 months before the baseline visit or during the duration of the study.

In mixed models corrected for longitudinal sampling, age at screening, and sex assigned at birth, SNAP-25 normalized protein expression (NPX) showed a strong positive association with MOG (estimate: 0.53 [0.32–0.73], $p < 0.001$), OMgp (0.10 [0.01–0.20], p = 0.034), GFAP (0.25 [0.09–0.41], p = 0.003), and CHI3L1 (0.16 [0.03–0.30], p = 0.017) (Figures 4A–4D). False discovery rate (FDR)-adjusted *p* values remained significant for MOG (0.005), GFAP (0.013), and CHI3L1 (0.023). Of note, those associations remained significant after correction for the degree of ongoing neuroaxonal injury, as assessed by neurofilament light chain (NfL) NPX (adjusted p = 0.009, 0.018, 0.027, for MOG, GFAP, and CHI3L1, respectively). sTREM2, CXCL-13, and CD27 did not correlate with SNAP-25 levels (p = 0.954, 0.314, and 0.233, respectively) (Figures 4E–4G). All *p* values were adjusted using the Benjamini-Hochberg approach. Our findings indicate a direct association between demyelination and synaptic loss, demonstrating that the latter is influenced, at least partially, by demyelination independent of axonal loss. The observed correlation with GFAP highlights the involvement of astrocytes in the process of synaptic loss and reinforces the predictive significance of GFAP in MS progression.

DISCUSSION

The pathological mechanism underlying progressive disability in MS remains unclear. Synaptic loss is an early feature underlying circuit dysfunction in MS as well as several neurodegenerative diseases. Here, we were able to (1) demonstrate that synaptic injury is an early event directly associated with downstream inflammatory demyelination in the retina of EAE, an animal model of MS; (2) document accelerated synaptic injury preceding disease progression in PwMS using an imaging-based approach that can measure synaptic volume in a single pathway; and (3) reveal a strong correlation between subclinical demyelination, aberrant glial activation, and synaptic loss independent of axonal damage using blood biomarkers in an independent cohort of PwMS.

A

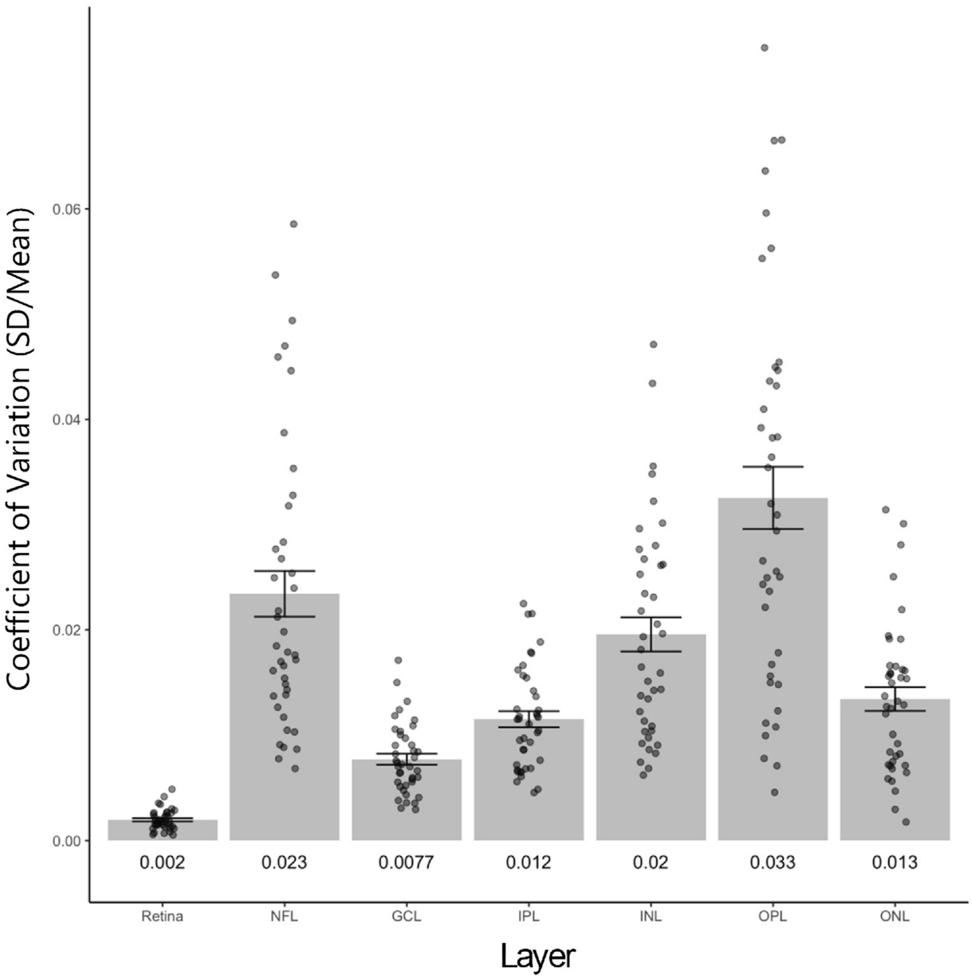

B

Layer	Average layer thickness Mean (SD)	Coefficient of variation Mean (SD)	Absolute mean deviation Mean (SD, range)	*Pearson's Correlation coefficient by image order* i12	i13	i14	i56	i57	i58
Retina	334.36 (10.77)	0.002 (0.001)	0.572 (0.45, 0.01 -1.81)	0.99	0.99	0.99	0.99	0.99	0.99
NFL	23.91 (1.89)	0.0234 (0.0138)	0.438 (0.39, 0 -1.92)	0.90	0.83	0.84	0.93	0.89	0.92
GCL	49.61 (2.6)	0.0077 (0.0033)	0.296 (0.19, 0.01 -0.67)	0.98	0.98	0.99	0.98	0.98	0.97
IPL	41.51 (1.79)	0.0115 (0.0048)	0.428 (0.33, 0.01 -1.38)	0.95	0.90	0.93	0.96	0.89	0.85
INL	34.96 (2.36)	0.0196 (0.0103)	0.635 (0.49, 0.08-2.15)	0.89	0.89	0.97	0.91	0.91	0.82
ONL	69.15 (4.75)	0.0134 (0.0071)	0.704 (0.55, 0.01 -2.02)	0.93	0.94	0.94	0.98	0.96	0.97
OPL	31.87 (4.04)	0.0325 (0.0186)	0.758 (0.64, 0.02 -2.62)	0.86	0.84	0.90	0.97	0.93	0.95

Figure 3. Retinal segmentation: CV by retinal layer

(A) Coefficient of variance (CV), defined as the intra-eye standard deviation (SD) divided by the intra-eye mean, by retinal sublayer. Bars represent mean CV; whiskers represent SE of the CV, and points represent eye-level data. Numeric text under each bar displays the mean CV for each layer.

(B) Group average thickness and intra-eye coefficient of variance, mean deviation, and Pearson's correlations of the segmentation repeatability imaging cohort for each retinal sublayer. Average cohort layer thickness, intra-eye CVs (SD/mean) and absolute mean deviations, and Pearson's correlation coefficients by image order for four images were acquired in sequence in each eye of 20 healthy controls in sequence. i12, comparison of the first and second image acquired; i13, first and third; i14 first and fourth; and so on. Images 1–4 were acquired in the right eye and 5–8 in the left.

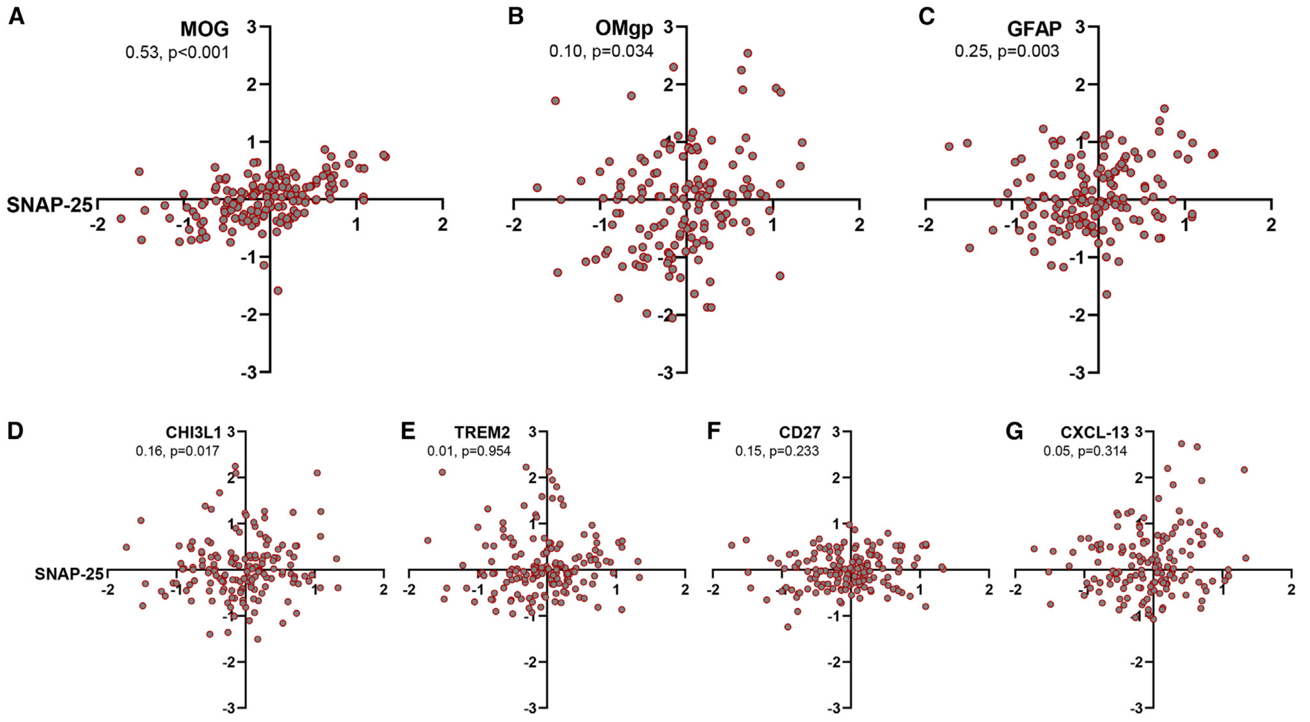

Figure 4. Hypothesis-driven analysis from a proteome-wide measurement performed on 166 samples from PwMS enrolled in the ReBUILD trial (Olink, Uppsala, Sweden). Relative protein concentration is reported as normalized protein expression (NPX) on a log-2 scale for each protein. Mixed models were corrected for longitudinal sampling, age at screening, and sex. Synaptosome-associated protein of 25 kDa (SNAP-25) NPX showed a strong positive association with myelin oligodendrocyte glycoprotein (MOG) (A), oligodendrocyte glycoprotein (OMgp) (B), glial fibrillary acidic protein (GFAP) (C), and chitinase 3-like 1 (CHI3L1) (D). False discovery rate (FDR)-adjusted *p* values remained significant for MOG (0.005), GFAP (0.013), and CHI3L1 (0.023). Soluble triggering receptor expressed on myeloid cells 2 (sTREM2) (E), CD27 (F), and chemokine ligand 13 (CXCL-13) (G) did not correlate with SNAP-25 levels.

The associated findings regarding the timing of IPL loss of synaptic density expand our knowledge regarding the timing of neuropathological events in EAE. Our results demonstrate that downstream demyelination (i.e., within the optic nerve) induces retinal synaptic loss.

Our finding that IPL thinning precedes and predicts the onset of SPMS provides an additional framework for understanding the biological basis for disability in MS. Together, these data support a model in which IPL synaptic injury is an early consequence of demyelination in EAE and heralds relentless progressive worsening. The accelerated synaptic loss in RR-SP, especially in the transition phase, aligns with the histopathological evidence of synaptic injury in MS.[13–15] Moreover, our data from animal models and using proteome-wide analysis strongly support the association between demyelination, glial involvement, and synaptic injury, regardless of neuroaxonal injury. This finding is in accordance with previous work describing synaptic loss as independent from axonal loss.[16]

Our results from serum biomarkers are supported by recent reports showing that higher blood MOG levels are associated with more severe clinical disease, worse cognitive function, and a higher burden of structural damage in MRI of PwMS.[46] Similarly, blood GFAP concentration could reflect and predict disease progression in PwMS,[47] highlighting the pathological role of astrocytes in disease progression. This aligns with translational work showing that astrocytes are involved in neurotoxicity, synaptic degeneration,[48] and dendrite removal during cell death.[49] The association of SNAP-25 levels with CHI3L1 could reflect either a role of toxic astrocytes or activated microglia in synaptic injury.[16] Unfortunately, the utilized Olink assays are currently not validated for use in mouse models and are not expected to perform well in mice at this stage. The antibodies included in the Olink assay are directed at human proteins. Standard LC/LC-MS proteomics have significant limitations for use on blood/serum/plasma and were therefore not considered in our study.

Indeed, microglia were separately found to be associated with what appears to be synaptic engulfment in the LGN,[16] and it has been shown in separate work to be increased in number (in EAE) 7 dpi in the retina.[34] This precedes the clinical onset of impairment and coincides with the time when the visual evoked potential N1 (the animal p100) latency delay is first detected.[32] Further evaluation of the relationship between microglial activation and injury and characterization of the precise molecular mechanisms driving this phenomenon are needed. On the contrary, B and T cell involvement markers were not associated with SNAP-25 levels in our study. It has been proposed that disability in progressive MS is driven mainly by innate, compartmentalized brain and spinal cord inflammation.[50–53] B and T cell activation markers in the blood are not necessarily associated with the

parenchymal/CSF inflammatory process.[7] Yet, those findings do not exclude a direct adaptive immune involvement in synaptic loss/progressive MS or an indirect effect mediated by aberrant glial activation.[54] Further studies evaluating CSF markers of immune cell activation and their association with synaptic damage are needed.

The results of this study support the potential use of IPL thickness as an endpoint for clinical trials. This work could also provide a rationale for further development of blood-based[55] and imaging[56] biomarkers of synaptic loss for disability progression in MS. Ongoing efforts are focused on optimizing the measurements of these proteins and addressing, at the tissue level, in different animal models of neurological conditions to what capacity they reflect CNS injury. Nevertheless, additional work will also be necessary to understand the timing of IPL change during the MS disease course and the potential effect of remyelination (and other possible treatments) on limiting and potentially reverse early synaptic injury. Ultimately, an integrated approach points toward an understanding of the pathological processes that tie adaptive immune-mediated demyelination to reductions in synaptic density, axonal loss, and permanent disability. This ongoing story is critical for framing a coherent understanding of the ways in which inflammatory demyelination leads to structural neuronal injury.

Limitations of the study

EAE is not a model of chronic progression in MS; for this reason, we were not able to study the association of IPL thinning with the progressive phase of the disease, and we focused on the timing of synaptic loss in the context of inflammatory demyelination. Also, the utilized Olink kits are not validated for use in mice. Olink Explore 3072 is fully validated for use in blood from MS patients but not in mice. Therefore, the reported biomarker association could not be replicated in EAE mice. To what degree the levels in the blood reflect the magnitude of corresponding tissue involvement remains incompletely defined for some of the included biomarkers. At this stage, Olink proteomics was performed on patients with stable RRMS. Future studies are needed to quantify SNAP-25 in patients presenting with a recent accumulation of disability.

STAR★METHODS

Detailed methods are provided in the online version of this paper and include the following:

- KEY RESOURCES TABLE
- RESOURCE AVAILABILITY
  - Lead contact
  - Materials availability
  - Data and code availability
- METHOD DETAILS
  - Study design
- EXPERIMENTAL MODEL AND SUBJECT DETAILS
  - Animal statement
  - Experimental autoimmune encephalomyelitis
  - Immunostaining of mouse retinal sections
  - Healthy control repeatability
  - MS participants
  - Retinal Imaging Protocol
  - Unbiased proteome-wide analysis in ReBUILD
- QUANTIFICATION AND STATISTICAL ANALYSIS
  - Image quantification and analysis

SUPPLEMENTAL INFORMATION

Supplemental information can be found online at https://doi.org/10.1016/j.xcrm.2024.101490.

ACKNOWLEDGMENTS

We want to thank all other UCSF MS EPIC Team investigators and staff: Jorge Oksenberg, Roland Henry, Sergio Baranzini, Michael Wilson, Riley Bove, Richard Cuneo, Sasha Gupta, Joseph Sabatino, Joanne Guo, Eduardo Caverzasi, Simone Sacco, Nico Papinutto, Jill Hollenbach, Jeff Gelfand, Sam Pleasure, Scott Zamvil, Douglas Goodin, Emmanuelle Waubant, Refujia Gomez, and Tiffany Cooper. This work was supported by National Institutes of Health grant R01NS105741 (to A.J.G. and C.C.), Italian Multiple Sclerosis Foundation (FISM) Rita-Levi Montalcini Grant code 2019/BC/002 (to C.C.), and the Dr. Miriam and Sheldon G. Adelson Medical Research Foundation (to J.R.C.).

The ReBUILD study was funded by generous support from The Rachleff Family Westridge Foundation and Janet Lustgarten and the Lustgarten Family Whitney Fund.

AUTHOR CONTRIBUTIONS

Conceptualization, A.J.G. and C.C.; methodology, A.J.G., C.C., S.W., A.A., and D.J.B.; investigation, C.C., S.W., A.A., D.J.B., A.B.-R., F.C.O., W.J.B., L.M., S.N., S.Y.C.M., K.A., A.B., J.H.S., I.P.S., and H.H.Y.; visualization, C.C., D.J.B., N.J., K.A., and J.R.C.; funding acquisition, A.J.G. and C.C.; project administration, A.J.G.; supervision, A.J.G., D.P.S., and J.R.C.; writing – original draft, A.J.G., C.C., J.R.C.; writing – review & editing, A.J.G., C.C., B.A.C.C., D.P.S., A.A., S.W., G.J.D., B.N., S.L.H., and J.R.C.

DECLARATION OF INTERESTS

The authors declare no competing interests.

Received: July 24, 2023
Revised: February 1, 2024
Accepted: March 12, 2024
Published: April 3, 2024

REFERENCES

1. Kappos, L., Wolinsky, J.S., Giovannoni, G., Arnold, D.L., Wang, Q., Bernasconi, C., Model, F., Koendgen, H., Manfrini, M., Belachew, S., and Hauser, S.L. (2020). Contribution of Relapse-Independent Progression vs Relapse-Associated Worsening to Overall Confirmed Disability Accumulation in Typical Relapsing Multiple Sclerosis in a Pooled Analysis of 2 Randomized Clinical Trials. JAMA Neurol. *77*, 1132–1140. https://doi.org/10.1001/jamaneurol.2020.1568.
2. Lublin, F.D., Häring, D.A., Ganjgahi, H., Ocampo, A., Hatami, F., Čuklina, J., Aarden, P., Dahlke, F., Arnold, D.L., Wiendl, H., et al. (2022). How patients with multiple sclerosis acquire disability. Brain *145*, 3147–3161. https://doi.org/10.1093/brain/awac016.
3. Abdelhak, A., Benkert, P., Schaedelin, S., Boscardin, W.J., Cordano, C., Oechtering, J., Ananth, K., Granziera, C., Melie-Garcia, L., Montes, S.C., et al.; MS EPIC, and the SMSC Study Teams (2023). Neurofilament Light Chain Elevation and Disability Progression in Multiple Sclerosis. JAMA Neurol. *80*, 1317–1325. https://doi.org/10.1001/jamaneurol.2023.3997.
4. Lassmann, H. (2018). Multiple Sclerosis Pathology. Cold Spring Harb. Perspect. Med. *8*, a028936. https://doi.org/10.1101/cshperspect.a028936.

5. Cree, B.A.C., Arnold, D.L., Chataway, J., Chitnis, T., Fox, R.J., Pozo Ramajo, A., Murphy, N., and Lassmann, H. (2021). Secondary Progressive Multiple Sclerosis: New Insights. Neurology *97*, 378–388. https://doi.org/10.1212/WNL.0000000000012323.
6. Mahad, D.H., Trapp, B.D., and Lassmann, H. (2015). Pathological mechanisms in progressive multiple sclerosis. Lancet Neurol. *14*, 183–193. https://doi.org/10.1016/S1474-4422(14)70256-X.
7. Lassmann, H. (2017). Targets of therapy in progressive MS. Mult. Scler. *23*, 1593–1599. https://doi.org/10.1177/1352458517729455.
8. Hauser, S.L., and Cree, B.A.C. (2020). Treatment of Multiple Sclerosis: A Review. Am. J. Med. *133*, 1380–1390.e2. https://doi.org/10.1016/j.amjmed.2020.05.049.
9. Lublin, F.D., Reingold, S.C., Cohen, J.A., Cutter, G.R., Sørensen, P.S., Thompson, A.J., Wolinsky, J.S., Balcer, L.J., Banwell, B., Barkhof, F., et al. (2014). Defining the clinical course of multiple sclerosis: the 2013 revisions. Neurology *83*, 278–286. https://doi.org/10.1212/WNL.0000000000000560.
10. Selkoe, D.J. (2002). Alzheimer's disease is a synaptic failure. Science *298*, 789–791. https://doi.org/10.1126/science.1074069.
11. Henstridge, C.M., Pickett, E., and Spires-Jones, T.L. (2016). Synaptic pathology: A shared mechanism in neurological disease. Ageing Res. Rev. *28*, 72–84. https://doi.org/10.1016/j.arr.2016.04.005.
12. Milnerwood, A.J., and Raymond, L.A. (2010). Early synaptic pathophysiology in neurodegeneration: insights from Huntington's disease. Trends Neurosci. *33*, 513–523. https://doi.org/10.1016/j.tins.2010.08.002.
13. Jürgens, T., Jafari, M., Kreutzfeldt, M., Bahn, E., Brück, W., Kerschensteiner, M., and Merkler, D. (2016). Reconstruction of single cortical projection neurons reveals primary spine loss in multiple sclerosis. Brain *139* (*Pt 1*), 39–46. https://doi.org/10.1093/brain/awv353.
14. Dutta, R., Chang, A., Doud, M.K., Kidd, G.J., Ribaudo, M.V., Young, E.A., Fox, R.J., Staugaitis, S.M., and Trapp, B.D. (2011). Demyelination causes synaptic alterations in hippocampi from multiple sclerosis patients. Ann. Neurol. *69*, 445–454. https://doi.org/10.1002/ana.22337.
15. Möck, E.E.A., Honkonen, E., and Airas, L. (2021). Synaptic Loss in Multiple Sclerosis: A Systematic Review of Human Post-mortem Studies. Front. Neurol. *12*, 782599. https://doi.org/10.3389/fneur.2021.782599.
16. Werneburg, S., Jung, J., Kunjamma, R.B., Ha, S.K., Luciano, N.J., Willis, C.M., Gao, G., Biscola, N.P., Havton, L.A., Crocker, S.J., et al. (2020). Targeted Complement Inhibition at Synapses Prevents Microglial Synaptic Engulfment and Synapse Loss in Demyelinating Disease. Immunity *52*, 167–182.e7. https://doi.org/10.1016/j.immuni.2019.12.004.
17. Green, A.J., McQuaid, S., Hauser, S.L., Allen, I.V., and Lyness, R. (2010). Ocular pathology in multiple sclerosis: retinal atrophy and inflammation irrespective of disease duration. Brain *133* (*Pt 6*), 1591–1601. https://doi.org/10.1093/brain/awq080.
18. Martinez-Lapiscina, E.H., Arnow, S., Wilson, J.A., Saidha, S., Preiningerova, J.L., Oberwahrenbrock, T., Brandt, A.U., Pablo, L.E., Guerrieri, S., Gonzalez, I., et al. (2016). Retinal thickness measured with optical coherence tomography and risk of disability worsening in multiple sclerosis: a cohort study. Lancet Neurol. *15*, 574–584. https://doi.org/10.1016/S1474-4422(16)00068-5.
19. Sotirchos, E.S., Gonzalez Caldito, N., Filippatou, A., Fitzgerald, K.C., Murphy, O.C., Lambe, J., Nguyen, J., Button, J., Ogbuokiri, E., Crainiceanu, C.M., et al. (2020). Multiple Sclerosis Visual System (IMSVISUAL) Consortium (2020). Progressive Multiple Sclerosis Is Associated with Faster and Specific Retinal Layer Atrophy. Ann. Neurol. *87*, 885–896. https://doi.org/10.1002/ana.25738.
20. Bischof, A., Caverzasi, E., Cordano, C., Hauser, S.L., and Henry, R.G. (2017). Advances in Imaging Multiple Sclerosis. Semin. Neurol. *37*, 538–545. https://doi.org/10.1055/s-0037-1607278.
21. Cellerino, M., Cordano, C., Boffa, G., Bommarito, G., Petracca, M., Sbragia, E., Novi, G., Lapucci, C., Capello, E., Uccelli, A., and Inglese, M. (2019). Relationship between retinal inner nuclear layer, age, and disease activity in progressive MS. Neurol. Neuroimmunol. Neuroinflamm. *6*, e596. https://doi.org/10.1212/NXI.0000000000000596.
22. Petzold, A., Balcer, L.J., Calabresi, P.A., Costello, F., Frohman, T.C., Frohman, E.M., Martinez-Lapiscina, E.H., Green, A.J., Kardon, R., Outteryck, O., et al. (2017). Retinal layer segmentation in multiple sclerosis: a systematic review and meta-analysis. Lancet Neurol. *16*, 797–812. https://doi.org/10.1016/S1474-4422(17)30278-8.
23. Gold, R., Linington, C., and Lassmann, H. (2006). Understanding pathogenesis and therapy of multiple sclerosis via animal models: 70 years of merits and culprits in experimental autoimmune encephalomyelitis research. Brain *129* (*Pt 8*), 1953–1971. https://doi.org/10.1093/brain/awl075.
24. University of California San Francisco MS-EPIC Team; Cree, B.A.C., Gourraud, P.A., Oksenberg, J.R., Bevan, C., Crabtree-Hartman, E., Gelfand, J.M., Goodin, D.S., Graves, J., Green, A.J., et al. (2016). Long-term evolution of multiple sclerosis disability in the treatment era. Ann. Neurol. *80*, 499–510. https://doi.org/10.1002/ana.24747.
25. University of California San Francisco MS-EPIC Team; Cree, B.A.C., Hollenbach, J.A., Bove, R., Kirkish, G., Sacco, S., Caverzasi, E., Bischof, A., Gundel, T., Zhu, A.H., et al. (2019). Silent progression in disease activity-free relapsing multiple sclerosis. Ann. Neurol. *85*, 653–666. https://doi.org/10.1002/ana.25463.
26. Cordano, C., Nourbakhsh, B., Devereux, M., Damotte, V., Bennett, D., Hauser, S.L., Cree, B.A.C., Gelfand, J.M., and Green, A.J. (2019). pRNFL as a marker of disability worsening in the medium/long term in patients with MS. Neurol. Neuroimmunol. Neuroinflamm. *6*, e533. https://doi.org/10.1212/NXI.0000000000000533.
27. Cordano, C., Yiu, H.H., Oertel, F.C., Green, A.J., University of California San Francisco MS-EPIC Team; Gelfand, J.M., Hauser, S.L., and Cree, B.A.C. (2021). Retinal INL Thickness in Multiple Sclerosis: A Mere Marker of Neurodegeneration? Ann. Neurol. *89*, 192–193. https://doi.org/10.1002/ana.25933.
28. Cordano, C., Yiu, H.H., Abdelhak, A., Beaudry-Richard, A., Oertel, F.C., and Green, A.J. (2022). Reply to "Interpretation of Longitudinal Changes of the Inner Nuclear Layer in MS. Ann. Neurol. *92*, 156. https://doi.org/10.1002/ana.26367.
29. Cordano, C., Nourbakhsh, B., Yiu, H.H., Papinutto, N., Caverzasi, E., Abdelhak, A., Oertel, F.C., Beaudry-Richard, A., Santaniello, A., Sacco, S., et al. (2022). Differences in Age-related Retinal and Cortical Atrophy Rates in Multiple Sclerosis. Neurology *99*, e1685–e1693. https://doi.org/10.1212/WNL.0000000000200977.
30. Sacco, S., Caverzasi, E., Papinutto, N., Cordano, C., Bischof, A., Gundel, T., Cheng, S., Asteggiano, C., Kirkish, G., Mallott, J., et al.; University of California, San Francisco MS-EPIC Team (2020). Neurite Orientation Dispersion and Density Imaging for Assessing Acute Inflammation and Lesion Evolution in MS. AJNR. Am. J. Neuroradiol. *41*, 2219–2226. https://doi.org/10.3174/ajnr.A6862.
31. Bischof, A., Papinutto, N., Keshavan, A., Rajesh, A., Kirkish, G., Zhang, X., Mallott, J.M., Asteggiano, C., Sacco, S., Gundel, T.J., et al. (2022). Spinal Cord Atrophy Predicts Progressive Disease in Relapsing Multiple Sclerosis. Ann. Neurol. *91*, 268–281. https://doi.org/10.1002/ana.26281.
32. Cordano, C., Sin, J.H., Timmons, G., Yiu, H.H., Stebbins, K., Guglielmetti, C., Cruz-Herranz, A., Xin, W., Lorrain, D., Chan, J.R., and Green, A.J. (2022). Validating visual evoked potentials as a preclinical, quantitative biomarker for remyelination efficacy. Brain *145*, 3943–3952. https://doi.org/10.1093/brain/awac207.
33. Cruz-Herranz, A., Dietrich, M., Hilla, A.M., Yiu, H.H., Levin, M.H., Hecker, C., Issberner, A., Hallenberger, A., Cordano, C., Lehmann-Horn, K., et al. (2019). Monitoring retinal changes with optical coherence tomography predicts neuronal loss in experimental autoimmune encephalomyelitis. J. Neuroinflammation *16*, 203. https://doi.org/10.1186/s12974-019-1583-4.
34. Cruz-Herranz, A., Oertel, F.C., Kim, K., Cantó, E., Timmons, G., Sin, J.H., Devereux, M., Baker, N., Michel, B., Schubert, R.D., et al. (2021). Distinctive waves of innate immune response in the retina in experimental

CellPress
OPEN ACCESS

autoimmune encephalomyelitis. JCI insight *6*, e149228. https://doi.org/10.1172/jci.insight.149228.

35. Mei, F., Lehmann-Horn, K., Shen, Y.A.A., Rankin, K.A., Stebbins, K.J., Lorrain, D.S., Pekarek, K., A Sagan, S., Xiao, L., Teuscher, C., et al. (2016). Accelerated remyelination during inflammatory demyelination prevents axonal loss and improves functional recovery. Elife *5*, e18246. https://doi.org/10.7554/eLife.18246.
36. Green, A.J., Gelfand, J.M., Cree, B.A., Bevan, C., Boscardin, W.J., Mei, F., Inman, J., Arnow, S., Devereux, M., Abounasr, A., et al. (2017). Clemastine fumarate as a remyelinating therapy for multiple sclerosis (ReBUILD): a randomised, controlled, double-blind, crossover trial. Lancet (London, England) *390*, 2481–2489. https://doi.org/10.1016/S0140-6736(17)32346-2.
37. Oyler, G.A., Higgins, G.A., Hart, R.A., Battenberg, E., Billingsley, M., Bloom, F.E., and Wilson, M.C. (1989). The identification of a novel synaptosomal-associated protein, SNAP-25, differentially expressed by neuronal subpopulations. J. Cell Biol. *109*, 3039–3052. https://doi.org/10.1083/jcb.109.6.3039.
38. Agliardi, C., Guerini, F.R., Zanzottera, M., Bianchi, A., Nemni, R., and Clerici, M. (2019). SNAP-25 in Serum Is Carried by Exosomes of Neuronal Origin and Is a Potential Biomarker of Alzheimer's Disease. Mol. Neurobiol. *56*, 5792–5798. https://doi.org/10.1007/s12035-019-1501-x.
39. Gerhards, R., Pfeffer, L.K., Lorenz, J., Starost, L., Nowack, L., Thaler, F.S., Schlüter, M., Rübsamen, H., Macrini, C., Winklmeier, S., et al. (2020). Oligodendrocyte myelin glycoprotein as a novel target for pathogenic autoimmunity in the CNS. Acta Neuropathol. Commun. *8*, 207. https://doi.org/10.1186/s40478-020-01086-2.
40. Linington, C., and Lassmann, H. (1987). Antibody responses in chronic relapsing experimental allergic encephalomyelitis: correlation of serum demyelinating activity with antibody titre to the myelin/oligodendrocyte glycoprotein (MOG). J. Neuroimmunol. *17*, 61–69. https://doi.org/10.1016/0165-5728(87)90031-2.
41. Abdelhak, A., Foschi, M., Abu-Rumeileh, S., Yue, J.K., D'Anna, L., Huss, A., Oeckl, P., Ludolph, A.C., Kuhle, J., Petzold, A., et al. (2022). Blood GFAP as an emerging biomarker in brain and spinal cord disorders. Nat. Rev. Neurol. *18*, 158–172. https://doi.org/10.1038/s41582-021-00616-3.
42. Kleinberger, G., Yamanishi, Y., Suárez-Calvet, M., Czirr, E., Lohmann, E., Cuyvers, E., Struyfs, H., Pettkus, N., Wenninger-Weinzierl, A., Mazaheri, F., et al. (2014). TREM2 mutations implicated in neurodegeneration impair cell surface transport and phagocytosis. Sci. Transl. Med. *6*, 243ra86. https://doi.org/10.1126/scitranslmed.3009093.
43. Im, J.H., Yeo, I.J., Park, P.H., Choi, D.Y., Han, S.B., Yun, J., and Hong, J.T. (2020). Deletion of Chitinase-3-like 1 accelerates stroke development through enhancement of Neuroinflammation by STAT6-dependent M2 microglial inactivation in Chitinase-3-like 1 knockout mice. Exp. Neurol. *323*, 113082. https://doi.org/10.1016/j.expneurol.2019.113082.
44. Cubas-Núñez, L., Gil-Perotín, S., Castillo-Villalba, J., López, V., Solís Tarazona, L., Gasqué-Rubio, R., Carratalá-Boscá, S., Alcalá-Vicente, C., Pérez-Miralles, F., Lassmann, H., and Casanova, B. (2021). Potential Role of CHI3L1+ Astrocytes in Progression in MS. Neurol. Neuroimmunol. Neuroinflamm. *8*, e972. https://doi.org/10.1212/NXI.0000000000000972.
45. Hintzen, R.Q., van Lier, R.A., Kuijpers, K.C., Baars, P.A., Schaasberg, W., Lucas, C.J., and Polman, C.H. (1991). Elevated levels of a soluble form of the T cell activation antigen CD27 in cerebrospinal fluid of multiple sclerosis patients. J. Neuroimmunol. *35*, 211–217. https://doi.org/10.1016/0165-5728(91)90175-7.
46. Jalaleddini, K., Jakimovski, D., Keshavan, A., McCurdy, S., Leyden, K., Qureshi, F., Ghoreyshi, A., Bergsland, N., Dwyer, M.G., Ramanathan, M., et al. (2024). Proteomic signatures of physical, cognitive, and imaging outcomes in multiple sclerosis. Ann. Clin. Transl. Neurol. Advance online publication. https://doi.org/10.1002/acn3.51996.
47. Meier, S., Willemse, E.A.J., Schaedelin, S., Oechtering, J., Lorscheider, J., Melie-Garcia, L., Cagol, A., Barakovic, M., Galbusera, R., Subramaniam, S., et al. (2023). Serum Glial Fibrillary Acidic Protein Compared With Neurofilament Light Chain as a Biomarker for Disease Progression in Multiple Sclerosis. JAMA Neurol. *80*, 287–297. https://doi.org/10.1001/jamaneurol.2022.5250.
48. Liddelow, S.A., Guttenplan, K.A., Clarke, L.E., Bennett, F.C., Bohlen, C.J., Schirmer, L., Bennett, M.L., Münch, A.E., Chung, W.S., Peterson, T.C., et al. (2017). Neurotoxic reactive astrocytes are induced by activated microglia. Nature *541*, 481–487. https://doi.org/10.1038/nature21029.
49. Damisah, E.C., Hill, R.A., Rai, A., Chen, F., Rothlin, C.V., Ghosh, S., and Grutzendler, J. (2020). Astrocytes and microglia play orchestrated roles and respect phagocytic territories during neuronal corpse removal in vivo. Sci. Adv. *6*, eaba3239. https://doi.org/10.1126/sciadv.aba3239.
50. Magliozzi, R., Howell, O., Vora, A., Serafini, B., Nicholas, R., Puopolo, M., Reynolds, R., and Aloisi, F. (2007). Meningeal B-cell follicles in secondary progressive multiple sclerosis associate with early onset of disease and severe cortical pathology. Brain *130* (*Pt 4*), 1089–1104. https://doi.org/10.1093/brain/awm038.
51. Howell, O.W., Reeves, C.A., Nicholas, R., Carassiti, D., Radotra, B., Gentleman, S.M., Serafini, B., Aloisi, F., Roncaroli, F., Magliozzi, R., and Reynolds, R. (2011). Meningeal inflammation is widespread and linked to cortical pathology in multiple sclerosis. Brain *134* (*Pt 9*), 2755–2771. https://doi.org/10.1093/brain/awr182.
52. Robinson, T., Abdelhak, A., Bose, T., Meinl, E., Otto, M., Zettl, U.K., Dersch, R., Tumani, H., Rauer, S., and Huss, A. (2020). Cerebrospinal Fluid Biomarkers in Relation to MRZ Reaction Status in Primary Progressive Multiple Sclerosis. Cells *9*, 2543. https://doi.org/10.3390/cells9122543.
53. Ahmed, S.M., Fransen, N.L., Touil, H., Michailidou, I., Huitinga, I., Gommerman, J.L., Bar-Or, A., and Ramaglia, V. (2022). Accumulation of meningeal lymphocytes correlates with white matter lesion activity in progressive multiple sclerosis. JCI insight *7*, e151683. https://doi.org/10.1172/jci.insight.151683.
54. Linnerbauer, M., Wheeler, M.A., and Quintana, F.J. (2020). Astrocyte Crosstalk in CNS Inflammation. Neuron *108*, 608–622. https://doi.org/10.1016/j.neuron.2020.08.012.
55. Oeckl, P., Anderl-Straub, S., Danek, A., Diehl-Schmid, J., Fassbender, K., Fliessbach, K., Halbgebauer, S., Huppertz, H.J., Jahn, H., Kassubek, J., et al. (2023). Relationship of serum beta-synuclein with blood biomarkers and brain atrophy. Alzheimers Dement. *19*, 1358–1371. https://doi.org/10.1002/alz.12790.
56. Becker, G., Dammicco, S., Bahri, M.A., and Salmon, E. (2020). The Rise of Synaptic Density PET Imaging. Molecules *25*, 2303. https://doi.org/10.3390/molecules25102303.
57. Krumbholz, M., Theil, D., Cepok, S., Hemmer, B., Kivisäkk, P., Ransohoff, R.M., Hofbauer, M., Farina, C., Derfuss, T., Hartle, C., et al. (2006). Chemokines in multiple sclerosis: CXCL12 and CXCL13 up-regulation is differentially linked to CNS immune cell recruitment. Brain *129* (*Pt 1*), 200–211. https://doi.org/10.1093/brain/awh680.
58. Polman, C.H., Reingold, S.C., Edan, G., Filippi, M., Hartung, H.P., Kappos, L., Lublin, F.D., Metz, L.M., McFarland, H.F., O'Connor, P.W., et al. (2005). Diagnostic criteria for multiple sclerosis: 2005 revisions to the "McDonald Criteria. Ann. Neurol. *58*, 840–846. https://doi.org/10.1002/ana.20703.
59. Petracca, M., Cordano, C., Cellerino, M., Button, J., Krieger, S., Vancea, R., Ghassemi, R., Farrell, C., Miller, A., Calabresi, P.A., et al. (2017). Retinal degeneration in primary-progressive multiple sclerosis: A role for cortical lesions? Mult. Scler. *23*, 43–50. https://doi.org/10.1177/1352458516637679.
60. Aytulun, A., Cruz-Herranz, A., Aktas, O., Balcer, L.J., Balk, L., Barboni, P., Blanco, A.A., Calabresi, P.A., Costello, F., Sanchez-Dalmau, B., et al. (2021). APOSTEL 2.0 Recommendations for Reporting Quantitative Optical Coherence Tomography Studies. Neurology *97*, 68–79. https://doi.org/10.1212/WNL.0000000000012125.
61. Tewarie, P., Balk, L., Costello, F., Green, A., Martin, R., Schippling, S., and Petzold, A. (2012). The OSCAR-IB consensus criteria for retinal OCT quality assessment. PLoS One *7*, e34823. https://doi.org/10.1371/journal.pone.0034823.

62. Caverzasi, E., Papinutto, N., Cordano, C., Kirkish, G., Gundel, T.J., Zhu, A., Akula, A.V., Boscardin, W.J., Neeb, H., Henry, R.G., et al. (2023). MWF of the corpus callosum is a robust measure of remyelination: Results from the ReBUILD trial. Proc. Natl. Acad. Sci. USA *120*, e2217635120. https://doi.org/10.1073/pnas.2217635120.

63. Abdelhak, A., Cordano, C., Boscardin, W.J., Caverzasi, E., Kuhle, J., Chan, B., Gelfand, J.M., Yiu, H.H., Oertel, F.C., Beaudry-Richard, A., et al. (2022). Plasma neurofilament light chain levels suggest neuroaxonal stability following therapeutic remyelination in people with multiple sclerosis. In Journal of neurology, neurosurgery, and psychiatry (Advance online publication), jnnp-2022-329221. https://doi.org/10.1136/jnnp-2022-329221.

64. Wik, L., Nordberg, N., Broberg, J., Björkesten, J., Assarsson, E., Henriksson, S., Grundberg, I., Pettersson, E., Westerberg, C., Liljeroth, E., et al. (2021). Proximity Extension Assay in Combination with Next-Generation Sequencing for High-throughput Proteome-wide Analysis. Mol. Cell. Proteomics *20*, 100168. https://doi.org/10.1016/j.mcpro.2021.100168.

STAR★METHODS

KEY RESOURCES TABLE

REAGENT or RESOURCE	SOURCE	IDENTIFIER
Antibodies		
mouse mAb α-Bassoon	Enzo Lifesciences	ADI-VAM-PS003-F; RRID: AB_11181058
rabbit pAb Homer1	Synaptic Systems	160003; RRID:AB_887730
guinea pig pAb α-VGluT1	Sigma	AB5905; RRID:AB_2301751
Alexa-fluorophore-conjugated secondary antibodies	Thermo Fisher Scientific	N/A
Biological samples		
Human serum sample	Green et al.[36]	N/A
Mice retina	This paper	N/A
Chemicals, peptides, and recombinant proteins		
MOG$^{35-54,57}$ peptide	Genemed Synthesis	N/A
Critical commercial assays		
Olink explorer kit	Olink	3072
Experimental models: Organisms/strains		
8-10-week-old female mice	Cruz-Herranz et al.[33]	C57BL/6J
Software and algorithms		
MATLAB	This paper	N/A
ImageJ	NIH	version 1.53f
Zen Black acquisition software	Zeiss	N/A

RESOURCE AVAILABILITY

Lead contact

Further information and requests for resources should be directed to and will be fulfilled by the lead contact, Ari J. Green (agreen@ucsf.edu).

Materials availability

All materials are available to any researcher for purpose of reproducing or extending the analysis.

Data and code availability

All data and materials used in the analysis are available to any researcher for purposes of reproducing or extending the analysis. The MATLAB script used in the manuscript is reported in the supplementary material.

METHOD DETAILS

Study design

The overall aim of the study was to determine whether synaptic injury in the retina is associated with progression in multiple sclerosis. The objective of the first portion of the study was to investigate the retina in EAE as a means to identify a biomarker that can be predictive of neuronal loss. The objective of the second portion of the study was to validate our findings in EAE, investigating if people with MS (PwMS) who develop SPMS show greater IPL loss, using macular volume OCT as a sign of pronounced synaptic pathology. The objective of the third portion of the study was to explore the pathophysiological mechanisms that might drive synaptic injury in PwMS, using a proteome-wide analysis.

EXPERIMENTAL MODEL AND SUBJECT DETAILS

Animal statement

All animals were maintained in barrier facilities on a 12-h light/dark cycle with food and water *ad libitum*. Experiments were conducted in compliance with the ARVO Statement for the Use of Animals in Ophthalmic and Vision Research, and all protocols were approved by the Institutional Animal Care and Use Committee (IACUC) of the University of California, San Francisco.

Experimental autoimmune encephalomyelitis

As previously described,[33,34] experimental autoimmune encephalomyelitis (EAE) was induced in 8-10-week-old female C57BL-6J mice by subcutaneous (s.c.) injection of 200 μg MOG_{35-55} peptide (Genemed Synthesis, San Antonio, TX) emulsified in complete Freund's Adjuvant (CFA) containing 0.2 mg *Mycobacterium tuberculosis* H37Ra into the flanks of both hindlimbs. CFA control animals received s.c. injections without MOG_{35-55} peptide. On the day of immunization and two days later, all mice were also administered 300 ng of pertussis toxin (List biological laboratories, Campbell, CA) intraperitoneally. Weights and clinical scores were checked and recorded regularly (score 0.5: distal tail limpness; score 1: complete tail limpness, score 1.5: limp tail and hindlimb weakness, score 2: mild hindlimb paresis; score 2.5: unilateral hindlimb paralysis; score 3: bilateral hindlimb paralysis; score 4: moribund; score 5: death). No control mice developed clinical symptoms. EAE and littermate control mice were sacrificed for subsequent analyses at the indicated time points at onset (day 12), peak (day 18), and chronic stages of the disease (day 60).

Immunostaining of mouse retinal sections

Mice were anesthetized and transcardially perfused with 0.1M phosphate buffer (PB), followed by 4% paraformaldehyde (PFA) in 0.1M PB. Eyes were dissected and post-fixed for 60 min at 4°C in 4% PFA before being equilibrated in 30% sucrose in 0.1M PB and embedded in an equal mixture of 30% sucrose in 0.1M PB and OCT compound (ThermoFisher Scientific, Waltham, MA, USA). 10 μm retinal cross-cryosections were blocked and permeabilized for 60 min at ambient temperature in 10% normal goat serum in 0.1M PB containing 0.3% Triton X-100 (Sigma, St. Louis, MO, USA). After blocking, sections were incubated with primary antibodies at ambient temperature overnight. The following primary monoclonal (mAb) and polyclonal (pAb) antibodies have been used: mouse mAb α-Bassoon (Enzo Lifesciences, ADI-VAM-PS003-F, 1:500), rabbit pAb Homer1 (Synaptic Systems, 160003, 1:1000), and guinea pig pAb α-VGluT1 (Sigma, AB5905, 1:2000). The next day, sections were probed with respective Alexa-fluorophore-conjugated secondary antibodies (ThermoFisher Scientific, Waltham, MA, USA) for 60 min at ambient temperature. After washing, sections were mounted using vectashield with DAPI (Vector Laboratories, Burlingame, CA, USA). Sections stained for Bassoon were incubated in Liberate Antibody Binding Solution (Polysciences Inc, Warrington, PA, USA) for 10 min at ambient temperature before blocking to retrieve antigen-binding sites.

Healthy control repeatability

All 320 images collected from 20 healthy controls (40 eyes) met OSCAR-IB criteria and were included in the analysis. Intraretinal sublayer segmentation was performed in a semi-automated fashion by a single, blinded grader (DB) using Heyex HRA viewing module 6.8.3.0 (Heidelberg Engineering, Heidelberg, Germany), and quantified with a 3.45mm circular parafoveal measurement grid manually centered on the fovea. Participants were a median age (range) of 35.65 (23.3–57.2) years, 40% male, and had a mean (SD) image quality of 36.54 (±2.36) dB, and a median (range) automated real-time tracking (ART) number of 70 (68–71).

Average intraretinal sublayer thicknesses were calculated by multiplying the regional thickness from each segment of the measurement grid by the segment's two-dimensional area.

The coefficient of variation was calculated by dividing the intra-eye standard deviation by the intra-eye mean. The average mean deviation was defined as the absolute difference between a single image value, and the intra-eye mean. Pearson's correlation coefficients were calculated based on image sequence order for each sequential image compared to the first image acquired within that eye. Analysis of variation was used to compare absolute mean deviations of all layers between the referenced and non-referenced scans, with layer as a covariate. All statistical analysis was performed using R version 3.6.3.

MS participants

Participants diagnosed according to 2005 McDonald criteria for MS[58] were enrolled in a large observational cohort study at the University of San Francisco, California (Multiple Sclerosis Genetics-Expressions, Proteomics, Imaging, Clinical (MS EPIC Study) EPIC, at the University of San Francisco, California (UCSF). From this population, those without a history of significant ophthalmologic disease or retinal surgery were eligible for annual retinal imaging between 2008 and 2020. Participants with stable relapsing-remitting (RRMS) disease course matched to patients that had transitioned to secondary progressive (SPMS) disease course following study enrollment and had at least two retinal imaging were selected for the present analysis. The UCSF institutional review board approved this study, and all participants provided informed consent.

For the test-retest analysis of IPL segmentation, an additional 20 healthy controls without a history of neurologic or ophthalmologic disease or retinal surgery were recruited internally from the UCSF Department of Neurology between December 2018 and April 2019. The study was approved by the UCSF institutional review board and all participants provided informed consent.

Retinal Imaging Protocol

Retinal images were acquired under scotopic conditions with a Heidelberg Spectralis spectral-domain OCT imaging device (Heidelberg Engineering, Heidelberg, Germany). OCT protocol was previously published.[18,59] Macular volume scans consisted of 19 horizontal B-scans centered on the fovea with a central fixation target and a 20 ° × 15 ° pattern size. For MS patients, two (2) macular volume scans were acquired sequentially in each eye at each study visit, one with automatic real-time tracking (ART) set to average 70 images per acquisition, and a second with ART set at 16. Target resolution was 1024 A-scans per B-scan (High Resolution), but

resolution was adjusted to 512 A-scans per B-scan (High Speed) at the technician's discretion in the case of a difficult acquisition. For all follow-up visits, images were acquired with positional referencing.

For healthy controls, four (4) high-resolution (1024 A-scans per B-scan), ART 70, macular volume scans were acquired sequentially from each eye at a single imaging session, for a total of 8 sequential images per participant. (Methods S3: Examples of segmented IPL of a single initial and follow-up B-scan from the test-retest analysis. Related to Retinal Imaging Protocol, STAR methods). The right eye was imaged first. B-scan positional referencing was enabled between the first image acquired and two (2) of the following images per eye, while referencing was disabled for one (1) of the remaining images. The order in which the single non-referenced image from each eye was collected (i.e., 2nd, 3rd or 4th) was assigned randomly based on total image order. Participants were asked to sit back from the camera between each image acquisition, and the camera was not switched off between image acquisitions. We followed the APOSTEL guidelines[60] (Data S4: APOSTEL Table. Related to Retinal Imaging Protocol, STAR methods) and the Oscar-IB[61] criteria for reporting OCT studies.

Unbiased proteome-wide analysis in ReBUILD

The ReBUILD trial (NCT02040298); a double-blind, randomised, placebo-controlled, within-groups comparison trial was conducted to evaluate the remyelinating potentials of clemastine fumarate.[36] 50 PwMS were randomised into two groups; the first group (G1) received daily clemastine fumarate for the first 90 days, followed by placebo for 60 days. In group 2 (G2), patients were initially treated with placebo for 90 days, followed by the active substance for 60 days. Visual evoked potentials (VEP) were conducted at each visit, including the screening visit. The primary outcome parameter was improvement of P100 latency following the initiation of clemastine. The study design included evaluation of relapse history, DMT treatment, EDSS assessment, focal MRI inflammatory metrics (FLAIR lesions), and myelin-specific MRI sequences[62] at each visit. Serum samples were collected from a subset of participants who consented to longitudinal blood sample collection at each study visit (baseline, month 1, month 3, month 5)[63] and stored at the local biobank at -80°C.

Unbiased blood proteomics analysis was conducted using the proximity extension assay (PEA) with the Olink explorer kit 3072 (Olink, Uppsala, Sweden) at Olink analytical facility (Boston, USA). In summary, Olink explorer kit utilizes compatible antibody pairs to measure 2947 unique protein over eight 384-plex panels.[64] Relative protein concentration was reported as normalized protein expression (NPX) on a log-2 scale for each protein.

Quality control (QC) was conducted for each sample and assay. Sample QC was assessed for each sample using the internal controls (incubation and Amplification controls) and count per sample. Internal controls NPX beyond ±0.3 NPX from the plate median or count per sample may not be less 500 counts resulting in sample warning. Assays measuring median NPX of three triplicates of negative control >5 SD from the predefined values set for each assay received assay warning. Datapoints receiving QC/or assay warning were not included in the final dataset used for the analysis. In addition, we performed a PCA analysis to identify samples with mean NPX >5 SD of average total samples NPX and samples with very high NPX were also excluded.

QUANTIFICATION AND STATISTICAL ANALYSIS

Image quantification and analysis

Relapsing-remitting MS to Pre-SPMS transition

A total of 75 EPIC participants who had transitioned to SPMS following enrollment as of April 2020 were screened for eligibility. 19 of these participants had at least two retinal imaging exam dates, at least 6 months apart, prior to July 1st of their year of transition, while 56 had fewer than two retinal imaging exams prior to transition and were excluded from analysis. The 19 eligible SPMS participants were matched to stable RRMS participants based on gender, disease duration within 6 years, and age within 9 years at the time of the first exam considered. The two (2) RRMS participants with the smallest difference in age and disease duration were selected for each SPMS participant, following a 2:1 RRMS:SPMS matching scheme, from a pool of 696 RRMS patients with longitudinal retinal imaging and stable disease as of April 2020. 658 RRMS patients were excluded, while 38 were selected as best matches. A second exam from each matching RRMS patient was selected arbitrarily as the next available exam date that was at least 1 year after the matched baseline exam.

A single, blinded technician (DB) selected the single best macular volume scan from each eye, based on resolution, luminance, ART setting, and signal-to-noise ratio, that met OSCAR-IB criteria[61] for analysis. Images from one eye of the SPMS cohort were excluded for failure to meet OSCAR-IB criteria. Intraretinal sublayer segmentation was performed in a semi-automated fashion using Heyex HRA viewing module 6.8.3.0 (Heidelberg Engineering, Heidelberg, Germany), and quantified with a 3.45mm circular parafoveal measurement grid manually centered on the fovea. Average intraretinal sublayer thicknesses were calculated by multiplying the regional thickness from each segment of the measurement grid by the segment's two-dimensional area. Annualized rate of change was calculated at the eye level by subtracting the average sublayer thickness from the first exam from the average sublayer thickness at the second exam and dividing by the time between them as a year fraction, assuming a 365.25 days-year.

For the primary analysis, participants taking fingolimod and their selected matches were excluded (possible subclinical macular edema) from the analysis, resulting in a primary cohort of 16 SPMS participants (31 eyes) and 32 RRMS participants (64 eyes), all Caucasian ethnic background. There were no patients on other S1P modulators included. Clinical macular edema was assessed for and excluded if present by an expert neuro-ophthalmologist (AJG). For the secondary analysis, all 19 SPMS (37 eyes) and 38

RRMS (76 eyes) were included. A repeated-measures ANOVA was constructed to model the effect of the group on annualized rate of sublayer change within the eye in the matched cohort, accounting for the random effect of the individual. Secondary analyses were performed to analyze the impact of previous optic neuritis, baseline age, gender, follow-up scan quality, final EDSS and disease-modifying treatment on IPL rate of change. When disease-modifying treatments were added to the analysis as a covariate, they were classified in tiers based on their efficacy, as already described.[29] All statistical analysis was performed using R version 3.6.3.

IPL segmentation

To measure the retinal thickness of mice, DAPI staining was performed using Vectashiels with DAPI mounting medium to label retinal nuclei. Stained sections were imaged with a Zeiss ApoTome microscope, and the thickness of the IPL and the RGC layers were segmented.

High-quality images (at least three for each mouse) with no IPL and retina disruption (evaluated by an operator blinded for animal conditions) were chosen. Briefly, images were uploaded in the Fiji software with the Bio-Format importer tool. After converting the files to PNG, the retina was rotated to a vertical position. The edges of the layers of interest were traced using the paintbrush tool (thickness = 1, white). Using the Brightness/Contrast tool in Fiji, the minimum brightness was increased until only the white lines remained visible. Then, maximal contrast was applied. The segmented image was then uploaded in MATLAB and automatically segmented using the code reported in the supplementary material (Data S1: MATLAB code for retinal segmentation, related to IPL segmentation, STAR methods).

Synapse density/number analyses

For the assessment of presynaptic inputs (Bassoon, VGluT1) and postsynaptic compartments (Homer 1), and for determining the colocalization of pre-and postsynaptic terminals, three to four stained cross-sections at the level of the optic disc from each sample containing the central retina were imaged on a Zeiss LSM700 laser scanning confocal microscope equipped with diode lasers (405 nm, 488 nm, 594 nm, 647 nm) and Zen Black acquisition software (Zeiss; Oberkochen, Germany). Two to three randomly chosen 63x fields of view within the central retina (up to 250 μm away from the optic disc) were acquired with 3 z stack steps at 0.46–1 μm spacing for each eye. The same settings were used to obtain images from all samples within one experiment, and data analyses were performed using ImageJ (NIH, version 1.53f) as described previously with some modifications.[10] First, a consistent threshold range was determined using sample images for each condition. All images were subjected to background subtraction, and then manual thresholding blinded to condition for each channel within one experiment was performed (IsoData segmentation method, 85–255). Then, single planes of the z-stacks (3 z-planes per field of view) were subjected to the same background subtraction and thresholding. Then, the retina's IPL was selected as the region of interest (ROI), and the total synapse area in the ROI was measured from the thresholded images using the analyze particles function of ImageJ. The image calculator tool was used to visualize colocalized pre- and postsynaptic puncta from the previously thresholded images to determine the area of structural synapses within the ROI. The analyze particle function was then used again to quantify the total area of colocalized signals in the ROI. Data from single z-planes was averaged for each z stack of each field of view, and the mean of all fields of view from one animal was determined to assess changes in synaptic densities.

Synaptic counts per field of view area were calculated for 12 EAE female mice and 9 sham immunized mice at onset (day 12, four EAE mice and three sham-immunized mice), the peak of disease (day 18, four EAE mice and three sham-immunized mice), and at a chronic stage (day 60, four EAE mice and three sham-immunized mice) from images localized in the proximity of the optic nerve head (Figure 3A). The number of structural synapses per IPL thickness was calculated by multiplying the average synapse density [colocalization of both VGlut1/Homer and Bassoon/Homer (Figure 3B–3I)] across fields of view for a given animal with the IPL thickness obtained from segmentation (Figure 3L– 3O). Two-tailed Student's t-test was used to determine statistical significance between groups regarding synaptic density. Statistical significance was expressed as *$p < 0.05$, **$p < 0.01$, ****$p < 0.0001$.

Cell Reports Medicine, Volume *5*

Supplemental information

Synaptic injury in the inner plexiform layer of the retina is associated with progression in multiple sclerosis

Christian Cordano, Sebastian Werneburg, Ahmed Abdelhak, Daniel J. Bennett, Alexandra Beaudry-Richard, Greg J. Duncan, Frederike C. Oertel, W. John Boscardin, Hao H. Yiu, Nora Jabassini, Lauren Merritt, Sonia Nocera, Jung H. Sin, Isaac P. Samana, Shivany Y. Condor Montes, Kirtana Ananth, Antje Bischof, UCSF MS EPIC Team, Bardia Nourbakhsh, Stephen L. Hauser, Bruce A.C. Cree, Ben Emery, Dorothy P. Schafer, Jonah R. Chan, and Ari J. Green

```
clc;    % Clear the command window.
close all;  % Close all figures (except those of imtool.)
clear;  % Erase all existing variables. Or clearvars if you want.
workspace;  % Make sure the workspace panel is showing.
format long g;
format compact;
fontSize = 20;
%===============================================================================
% Read in a standard MATLAB gray scale demo image.
folder = pwd;
%===========================================================1
%===========================================================1
%===========================================================1
%===========================================================1
%=====================|| PLEASE EDIT ONLY THE LINE BELOW! ======================================1
%========================
%===========================================================1
%===========================================================1
NameOfImage = ['IPL_central_Retina_1a_02'];        % WRITE THE NAME OF THE IMAGE!
%===========================================================1
%===========================================================1
%===========================================================1
%===========================================================1
%===========================================================1
%===========================================================1
%===========================================================1
%===========================================================1
%===========================================================1
%===========================================================1
%===========================================================1
%===========================================================1
%===========================================================1
baseFileName = strcat(NameOfImage,'.png');
% Get the full filename, with path prepended.
fullFileName = fullfile(folder, baseFileName);
% Check if file exists.
if ~exist(fullFileName, 'file')
 % The file doesn't exist -- didn't find it there in that folder.
 % Check the entire search path (other folders) for the file by stripping off the folder.
 fullFileNameOnSearchPath = baseFileName; % No path this time.
 if ~exist(fullFileNameOnSearchPath, 'file')
   % Still didn't find it.  Alert user.
   errorMessage = sprintf('Error: %s does not exist in the search path folders.', fullFileName);
   uiwait(warndlg(errorMessage));
   return;
 end
end
grayImage = imread(fullFileName);
% Get the dimensions of the image.
% numberOfColorChannels should be = 1 for a gray scale image, and 3 for an RGB color image.
[rows, columns, numberOfColorChannels] = size(grayImage);
if numberOfColorChannels > 1
 % It's not really gray scale like we expected - it's color.
 % Use weighted sum of ALL channels to create a gray scale image.
 grayImage = rgb2gray(grayImage);
 % ALTERNATE METHOD: Convert it to gray scale by taking only the green channel,
 % which in a typical snapshot will be the least noisy channel.
 % grayImage = grayImage(:, :, 2); % Take green channel.
end
% Display the image.
subplot(2, 2, 1);
imshow(grayImage);
title('Original Grayscale Image', 'FontSize', fontSize, 'Interpreter', 'None');
% Set up figure properties:
% Enlarge figure to full screen.
set(gcf, 'Units', 'Normalized', 'OuterPosition', [0, 0, 1, 1]);
% Get rid of tool bar and pulldown menus that are along top of figure.
% set(gcf, 'Toolbar', 'none', 'Menu', 'none');
% Give a name to the title bar.
set(gcf, 'Name', 'IPL Segmentation', 'NumberTitle', 'Off')
% Get a binary image of the black lines
binaryImage = grayImage > 128;
[labeledImage, numBlobs] = bwlabel(binaryImage);
% Display the image.
```

```
subplot(2, 2, 2);
imshow(binaryImage, []);
axis on;
title('Binary Image', 'FontSize', fontSize, 'Interpreter', 'None');
% Find width at every row
widths = zeros(rows, 1);
for row = 1 : rows
 thisRow = binaryImage(row, :);
 leftPixel = find(thisRow, 1, 'first');
 rightPixel = find(thisRow, 1, 'last');
 if isempty(leftPixel) || isempty(rightPixel)
   continue; % Skip lines where there is no white pixel.
 end
widths(row) = rightPixel - leftPixel; % Add 1 if you want whole pixels instead of pixel center-to-pixel
center.
end
% Display the plot.
subplot(2, 2, 3:4);
plot(widths, 'b-', 'LineWidth', 2);
grid on;
xlabel('Row', 'FontSize', fontSize);
ylabel('Width in pixels', 'FontSize', fontSize);
title('Widths', 'FontSize', fontSize);

T = table(widths);
B = rmoutliers(T, 'mean');
ResultsTable = strcat(NameOfImage,'.xlsx');
writetable(B, ResultsTable);
```

Data S1: **MATLAB code for retinal segmentation**, related to IPL segmentation, STAR methods.

Cohort				
	**RR-RR**	**RR-SP**	**p**	**Standardized Mean Difference**
n *patients*	38	19		
n *eyes*	76	37		
Fingolimod treated *N eyes, (%)*	4 (5.3)	6 (16.2)	0.026	0.109
Male sex *N eyes, (%)*	40 (52.6)	20 (54.1)	1	0.029
Baseline Age (years) *mean (SD)*	47.68 (10.17)	48.28 (10.48)	0.773	0.058
Baseline Disease Duration (years) *median [IQR]*	13.25 [9.70, 20.20]	14.30 [7.80, 19.60]	0.845	0.03
Final EDSS *median [IQR]*	2.00 [1.50, 3.00]	4.50 [3.375, 6.00]	<0.001	1.927
Study Interval (years) *mean (SD)*	3.80 (1.58)	3.43 (2.16)	0.297	0.198
Baseline Image Quality (dB) *mean (SD)*	33.42 (3.35)	33.16 (4.64)	0.736	0.064
Baseline IPL Thickness (µm) *mean (SD)*	37.00 (5.15)	35.64 (4.07)	0.16	0.294
Baseline Retinal Thickness (µm) *mean (SD)*	324.42 (18.98)	321.81 (13.93)	0.459	0.157

Data S2: **Demographics and imaging information for the full cohort**. Related to Retinal Imaging Protocol, STAR methods. Demographics, baseline age, baseline disease duration, final EDSS, study interval, image quality, baseline inner plexiform layer (IPL), and baseline retinal thickness for patients with stable relapsing-remitting disease (RR-RR) and patients who transitioned to secondary progressive on study (RR-SP). Mean and Standard deviation (SD) are represented for normally distributed variables, while median and Interquartile range (IQR) and represented for non-normally distributed variables. P values are derived from students t-test for normally distributed continuous variables, Wilcoxon-Rank Sum test for non-normally distributed variables, and Fisher's Exact test for categoric variables.

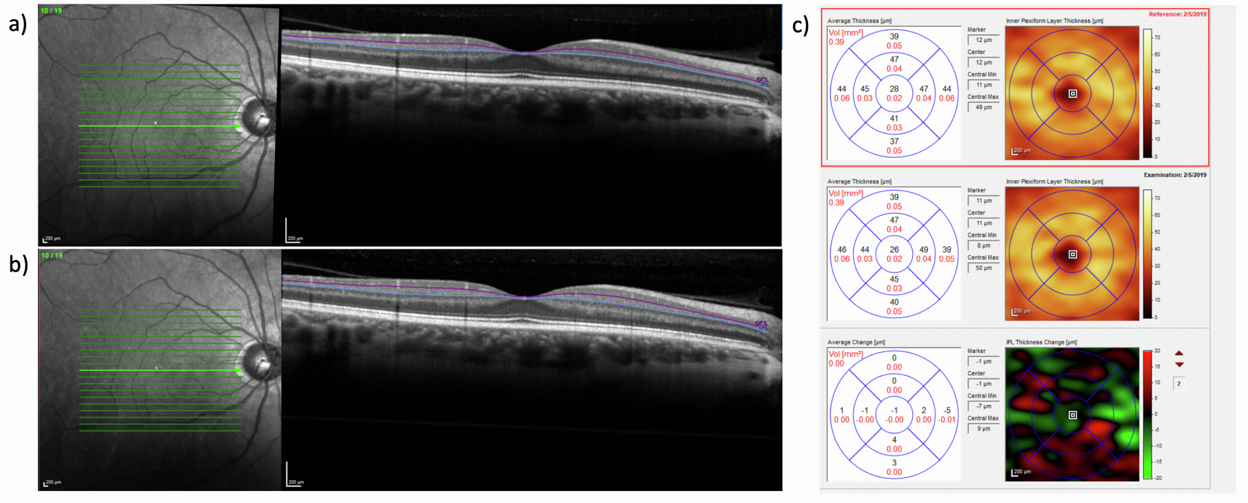

<u>Methods S3:</u> **Examples of segmented IPL of a single initial (a) and follow-up (b) B-scan from the test-retest analysis.** Related to Retinal Imaging Protocol, STAR methods. Panel C shows IPL thickness heatmaps across the full macular fundus, with measurement grids displaying calculated IPL volumes and the difference in measurement between the initial and follow-up volume scans.

Inclusion criteria & Screening	Inclusion criteria	The inclusion criteria for this study are those of the parent observational study EPIC (cite). Briefly, patients qualified for EPIC if they met diagnostic criteria for CIS or multiple sclerosis. The entire EPIC cohort was screened for inclusion in this retrospective analysis. SPMS participants were selected for inclusion if they had transitioned from RRMS to SPMS following study enrollment, and had at least 2 OCT exams prior to July 1st of the year of clinical transition. A group of participants who have remained RRMS while on study were matched to participants in the SPMS group by gender, disease duration within 6 years, and age within 9 years at time of first OCT exam considered. This was done in a 2:1 control:patient matching scheme. For the primary analysis, participants were excluded if they had taken Fingolimod during the study period considered. For the secondary analysis, participants were included irrespective of Fingolimod history. At the eye level, exclusion criteria included evidence of ophthalmologic disease and failure to meet OSCARIB quality guidelines.
	Total participants screened	75 SPMS patients (all those who had transitioned on study as of Apr. 2020), 696 RRMS patients with stable disease
	SPMS Participants Excluded (reason for exclusion criteria)	56<br>(Less than 2 OCT exam dates prior to year of transition)
	Participants Excluded (reason for exclusion criteria)	658<br>(Only the 2 closest matches to the gender, age and disease duration of a SPMS patient were selected)
	Primary Analysis Participants Included (Eyes included)	16 RR-SP (31 eyes), 32 RR-RR (64 eyes)
	Secondary Analysis Participants Included (Eyes included)	19 RR-SP (37 eyes), 38 RR-RR (76 eyes)
Image Acquisition	Protocol	14 trained technicians acquired OCT images under scotopic conditions without pharmacologic dilation between Oct. 29, 2008 and Sept. 26, 2018. At each exam, 2 or more rings scans, 1 radial scan, 2 macular volume scans, and 1 posterior pole scans were acquired with the target parameters shown below. If imaging was not possible with these settings, images were acquired with reduced ART number and/or with high speed resolution settings.
	Device	Heidelberg Spectralis OCT (Model Code S2400), Heideleberg Engineering, Heideleberg, Germany
	Wavelength	870 nm
	Camera Objective	Standard
	**Acquisition Software Versions**	**Date range used (N exams)**
	3.2.1.0	10/29/2008-10/29/2008 (1)
	3.2.1.5	11/07/2008-12/01/2008 (2)
	4.0.0.0	12/08/2008-12/02/2009 (8)
	5.0.2.0	04/16/2009-11/10/2009 (7)
	5.1.2.0	12/11/2009-02/23/2010 (2)
	5.2.4.0	11/10/2010-11/10/2010 (1)
	5.3.3.0	03/16/2011-12/01/2011 (4)
	5.4.7.0	03/19/2012-08/05/2015 (48)
	5.6.3.0	05/07/2014-05/07/2014 (1)
	5.6.4.0	04/08/2013-06/25/2013 (2)
	5.7.5.0	10/23/2014-10/30/2014 (2)
	6.0.10.0	01/22/2015-08/10/2015 (6)
	6.0.13.0	08/24/2015-09/26/2018 (42)

Target Parameters

Scan Type	Anatomical Structure	Fixation Target Location	Eye Tracking	images acquired	Target Resolution	Target ART Number	Number of B-Scans, Orientation	Scan Angle (degrees)	Pattern Size (degrees)	A-scans per B-scan (HR/HS)	Depth
Ring	pRNFL	Nasal	On	>2	High Resolution	100	1 Circular	12	12 (diameter)	1536/768	1.9mm
Radial	ONH	Nasal	On	1	High Resolution	25	25 Radial	15	15 (7.5 between B-scans)	768/384	1.9mm
Macular Volume	Parafoveal macula	Middle	On	2	High Resolution	70, 16	19 Horizontal	20	20x15	1024/512	1.9mm
Posterior Pole Volume	papillomacular bundle	Middle	On	1	High Speed	10	61 Horizontal	30	30x25	768 (HS Only)	1.9mm

Post-acquisition Analysis	Image selection	The single best (highest signal strength, ART number, and resolution setting) macular volume scan meeting OSCAR-IB quality criteria per eye and exam date was elected by a single, masked grader
	Images reviewed N	RR-SP: 351, RR-RR: 706
	Primary Analysis Images selected N (% of total reviewed)	RR-SP: 62 (18%), RR-RR: 128 (18%)
	Secondary Analysis Images selected N (% of total reviewed)	RR-SP: 74 (21%), RR-RR: 152 (22%)
	Primary Analysis High Speed Images selected N (% of total selected)	RR-SP: 6 (10%), RR-RR: 2 (2%)
	Secondary Analysis High Speed Images selected N (% of total selected)	RR-SP: 13 (18%), RR-RR: 2 (1%)
	Primary Analysis ART number of selected images median (range)	RR-SP: 68 (14-71), RR-RR: 70 (15-71)
	Secondary Analysis ART number of selected images median (range)	RR-SP: 68 (14-71), RR-RR: 70 (15-71)
	Primary Analysis Signal strength	RR-SP: 32 (5), RR-RR: 33 (3)

	(Quality) of selected images mean (SD)	
	Secondary Analysis Signal strength (Quality) of selected images mean (SD)	RR-SP: 32 (5), RR-RR: 33 (3)
	**Segmentation**	All segmentation was performed in a semi-automated fashion by a single, masked grader
	**Segmentation software**	Heyex HRA Viewing Module 6.8.3.0, Heidelberg Engineering, Heidelberg, Germany
	**Grid used for data extraction**	1, 2.22, 3.45mm circular measurement grid centered on fovea. All regions used.
	**Target structures** (units)	Intraretinal sub layers (thickness, um)
	**Statistical Analysis**	
	**Averaging/Eye selection strategy**	Average intraretinal layer thickness was calculated by multiplying regional thickness from each segment of the measurement grid by the segment's 2-dimensional area. Annualized rate of change was calculated at the eye level subtracting average thickness at the first exam, from average thickness at the second exam, and dividing by the year fraction (assuming a 365.25 day year) between the two exams. Each eye meeting OSCARIB criteria at both exams was included in analysis (the random effect of individual was included in statistical modeling to correct for violation of independence)
	**Statistical method**	Repeated measures ANOVA, Repeated measures ANCOVA

Data S4: **APOSTEL Table**. Related to Retinal Imaging Protocol, STAR methods.
